# Attitudes Toward Artificial Intelligence Within Dermatopathology: An International Online Survey

**DOI:** 10.3389/fmed.2020.591952

**Published:** 2020-10-20

**Authors:** Sam Polesie, Phillip H. McKee, Jerad M. Gardner, Martin Gillstedt, Jan Siarov, Noora Neittaanmäki, John Paoli

**Affiliations:** ^1^Department of Dermatology and Venereology, Institute of Clinical Sciences, Sahlgrenska Academy, University of Gothenburg, Gothenburg, Sweden; ^2^Region Västra Götaland, Sahlgrenska University Hospital, Department of Dermatology and Venereology, Gothenburg, Sweden; ^3^Retired, Boston, MA, United States; ^4^Department of Laboratory Medicine, Geisinger Medical Center, Danville, PA, United States; ^5^Department of Pathology, Institute of Biomedicine, Sahlgrenska Academy, University of Gothenburg, Gothenburg, Sweden

**Keywords:** artificial intelligence, attitude, dermatopathology, machine learning, online survey

## Abstract

**Background:** Artificial intelligence (AI) has recently surfaced as a research topic in dermatology and dermatopathology. In a recent survey, dermatologists were overall positive toward a development with an increased use of AI, but little is known about the corresponding attitudes among pathologists working with dermatopathology. The objective of this investigation was to make an inventory of these attitudes.

**Participants and Methods:** An anonymous and voluntary online survey was prepared and distributed to pathologists who regularly analyzed dermatopathology slides/images. The survey consisted of 39 question divided in five sections; (1) AI as a topic in pathology; (2) previous exposure to AI as a topic in general; (3) applications for AI in dermatopathology; (4) feelings and attitudes toward AI and (5) self-reported tech-savviness and demographics. The survey opened on March 13, 2020 and closed on May 5, 2020.

**Results:** Overall, 718 responders (64.1% females) representing 91 countries were analyzed. While 81.5% of responders were aware of AI as an emerging topic in pathology, only 18.8% had either good or excellent knowledge about AI. In terms of diagnosis classification, 42.6% saw strong or very strong potential for automated suggestion of skin tumor diagnoses. The corresponding figure for inflammatory skin diseases was 23.0% (P_adj_ < 0.0001). For specific applications, the highest potential was considered for automated detection of mitosis (79.2%), automated suggestion of tumor margins (62.1%) and immunostaining evaluation (62.7%). The potential for automated suggestion of immunostaining (37.6%) and genetic panels (48.3%) were lower. Age did not impact the overall attitudes toward AI. Only 6.0% of the responders agreed or strongly agreed that the human pathologist will be replaced by AI in the foreseeable future. For the entire group, 72.3% agreed or strongly agreed that AI will improve dermatopathology and 84.1% thought that AI should be a part of medical training.

**Conclusions:** Pathologists are generally optimistic about the impact and potential benefit of AI in dermatopathology. The highest potential is expected for narrow specified tasks rather than a global automated suggestion of diagnoses. There is a strong need for education about AI and its use within dermatopathology.

## Introduction

Even though artificial intelligence (AI) has been discussed for decades, the unprecedented computer processor development, storage availability, and universal internet access including cloud services have contributed to the recent accelerated research interest in AI in several domains of medicine including pathology (1). AI and machine learning (ML) have recently pervaded every aspect of medical image analysis at an extraordinary pace (2).

While historically restricted to traditional microscopes and glass slides, pathology has recently transitioned to become a more digitally oriented specialty. Within the past decade, the use of whole slide imaging (WSI) has been validated for surgical pathology and dermatopathology (3, 4). Although WSI has not uniformly been well-received among dermatopathologists (5), the diagnostic modality has proven non-inferior to traditional microscopes for most diagnostic classes, except for a subset of melanocytic lesions, in a recent intra-observer validation investigation (6). With the rise of whole slide scanner technology (7), and free-hand smartphone photography (8), large numbers of tissue slides are being captured and archived digitally. For dermatopathology specifically, closed online forums now represent an important piece of continuing medical education and attract thousands of users from multiple countries across the world in all age groups (9). In many centers, all glass slides are routinely scanned and are made available digitally. While the primary diagnosis might still be made by pathologists using traditional microscopes, ancillary diagnoses by ML-based software might only be a few computer maneuvers away, fundamentally changing pathology as we know it.

The immense accumulation of online dermatopathology images represents a substantial resource not only for education, but also for ML research purposes. Recently, Schaumberg et al. presented the first pan-tissue and pan-disease ML-method for predicting disease as well as immunostaining (10). The training set consisted of thousands of images obtained through social media and PubMed articles and was prospectively tested in public on social media. Although in its early stages, the potential seen for ML in dermatopathology have been demonstrated both pertaining to specific tasks as well as diagnosis (11–13). The use of ML within dermatopathology has recently been listed as one of the important dermatological applications of AI (14). Nonetheless, little is known about dermatopathologists' attitudes toward an increased use of AI within their field. In a recent study, including 1,271 dermatologists, investigating the attitudes toward AI within dermatology, the majority of responders were positive toward a development with an increased use of AI. Moreover, only a minority expressed fear toward an increased use. Interestingly, a majority of responders (58.1%) saw either strong or very strong potential for automated detection of skin diseases based on dermatopathology images (15).

Dermatopathology and dermatology depend on one another and collaboration between our disciplines is essential for clinical-pathological correlation. In order to prepare for a future with an increased use of AI, it is our belief that a first inventory of dermatopathologists' views on the topic is important. Ultimately, it is our hope that dermatopathologists together with dermatologists should be the ones who guide and lead the development in our fields. Thus, the following study was performed as an exploratory survey with the aim to specifically address feelings and attitudes toward AI among dermatopathologists.

## Materials and Methods

An anonymized and voluntary English online survey was prepared and distributed through SurveyMonkey® (www.surveymonkey.com, San Mateo, CA, USA). The survey started with a brief introduction followed by 39 questions (Q1–Q39). The first baseline question (Q1) tested for eligibility (i.e., if the responders worked as a pathologist who regularly analyzed dermatopathology slides/images). If a responder answered “no” to this first question, the survey ended. The rest of the survey questions were divided into five sections: (1) AI as a topic in pathology (Q2-Q3); (2) previous exposure to AI as a topic in general (Q4-Q7); (3) applications for AI in dermatopathology (Q8-Q14); (4) feelings and attitudes toward AI (Q15-Q25) and (5) self-reported tech-savviness and demographics (Q26-Q39). Most of the response options were prepared as Likert-type scales (16). The complete survey as displayed to the responder is available in the Supplementary Material. Answers to all questions were required for the survey to be considered complete.

The survey opened on March 13, 2020 and closed on May 5, 2020 (53 survey days). The survey link was advertised on a closed Facebook® (Menlo Park, CA, USA) group called McKee Derm, which is frequently visited by international pathologists, dermatopathologists and dermatologists and has ~15,000 members (17). The group opened in May 2017 and its main purpose is to share and discuss dermatopathology cases. The survey link was also shared publicly by one of the authors (JMG) on their public Facebook page and Twitter account. Since all responses were obtained voluntarily and anonymously from professional colleagues, the study was exempt from approval by an ethics review board.

### Statistical Analysis

All data were analyzed using R version 3.5.3 (https://www.r-project.org/). Fisher's exact test and Wilcoxon's rank sum test were used for two-sample tests. Wilcoxon's signed rank test was used for paired tests. For questions with dichotomous answers, logistic regression was used with “*no*”*/*“*yes*” as the dependent variable and sex and age group as independent variables. For questions with non-dichotomous answers, linear regression models were used to correlate answers to sex and age group using a score for the answers, for example: “*Strongly disagree”* = 1, “*Disagree”* = 2, “*Neither agree nor disagree”* = 3, “*Agree”* = 4 and “*Strongly agree”* = 5. The age groups (20-24, 25-34, 35-44, 45-54, 55-64, 65-74, and ≥75 years) were used as numeric values in the regression models, i.e., numbers ranging from 1 to 7. P-values were adjusted (*P*_*adj*_) for multiple comparisons using the Holm method (18). All tests were two-sided and *P*_*adj*_ < 0.05 was considered as statistically significant.

## Results

**Figure 1:**
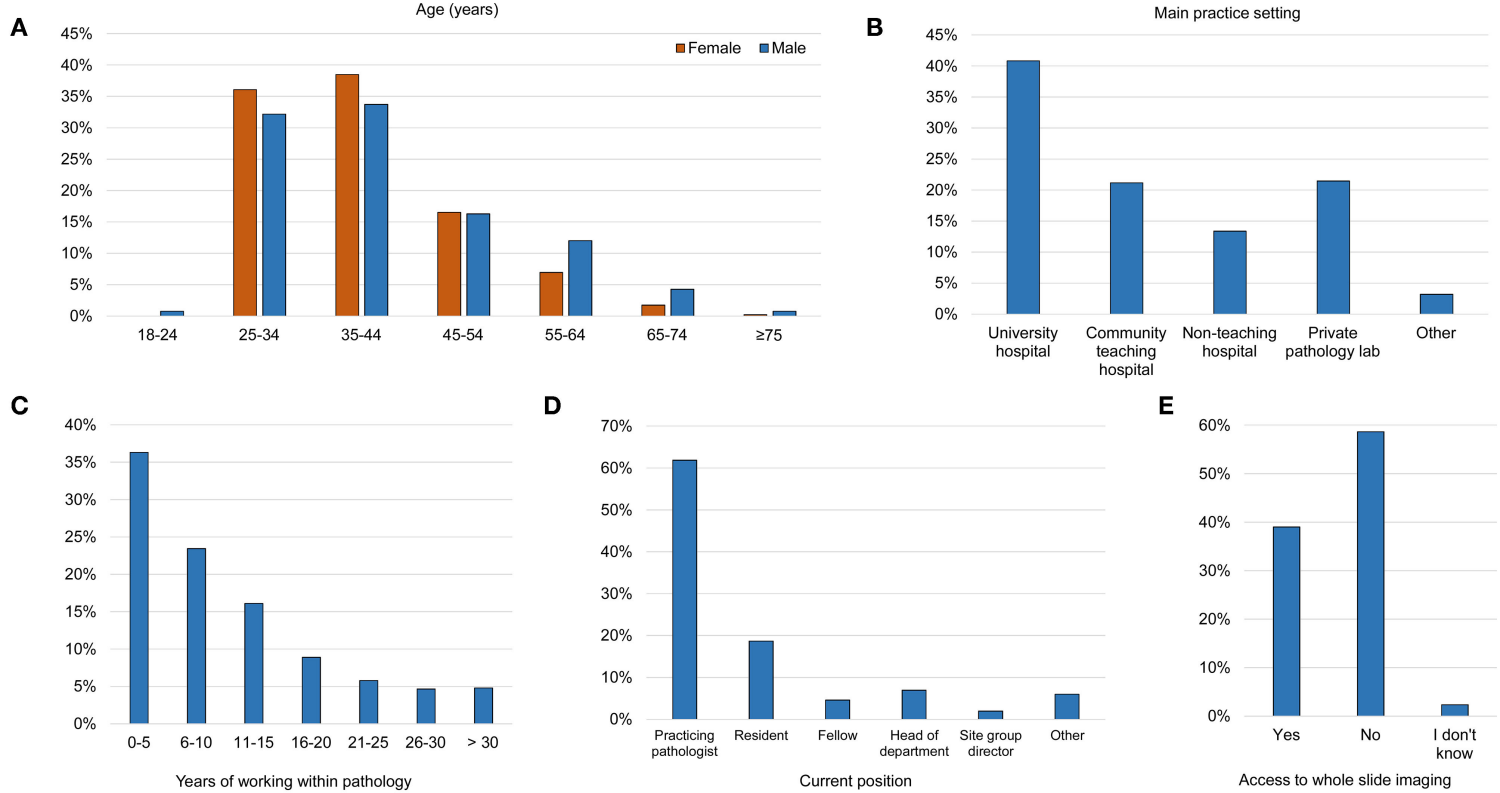
Responder characteristics. **(A)** Age distribution for each sex. **(B)** Proportion in each type of practice setting. **(C)** Distribution of the number of years working within pathology. **(D)** Current position. **(E)** Access to whole slide imaging at work.

Of 1,263 surveys received, 848 responders worked as pathologists who regularly analyzed dermatopathology slides/images in a clinical setting. Among these, 718 completed the survey resulting in an 84.7% response rate among the eligible audience and these responses were further analyzed. The median time to complete the survey was 6.1 min (interquartile range, 4.7–8.5 min). All aggregated result summaries for Q1-Q38 are available in Supplementary Material. An overview of the age and sex distribution of the responders as well as other demographic data are presented in Figure 1. Practicing specialists and residents in pathology constituted 80.5% of the responders. The median age (range) was 38 years (22–79 years), 64.1% (*n* = 460) were females and 39.0% (*n* = 280) had access to WSI at work. The great majority of the surveyed dermatopathologists mainly used traditional microscopes for routine diagnostic dermatopathology while only 4% (29 of 718) of the responders mainly used digitally scanned slides. Overall, 91 different countries were represented. The three countries where most responders worked were India (*n* = 96, 13.4%), USA (*n* = 80, 11.1%) and United Kingdom (*n* = 35, 4.9%).

While 81.5% of responders were aware of AI as an emerging topic in pathology, only 18.8% had either good or excellent knowledge about AI within pathology. Younger responders (<40 years) were equally aware of the recent development compared to older responders (≥40 years) with 322 of 400 (80.5%) vs. 263 of 318 (82.7%) claiming awareness (*P* = 0.50, *P*_*adj*_ = 1). A higher percentage of responders mainly working in a university hospital (255 of 293, 87.0%) were aware of the AI development within pathology compared to those working in other settings (330 of 425, 77.6%) (*P* = 0.0017, *P*_*adj*_ = 0.073). Males generally had a higher self-reported level of knowledge about AI than females (26.0 vs. 14.8%, *P* = 0.003, *P*_*adj*_ = 0.12).

Among all responders, 22.3% had used AI as a diagnostic aid in real life within pathology and 11.0% had used AI within dermatopathology in particular. Use of AI was not associated with age nor sex (Table 1). Physicians with access to WSI and those with previous use of AI within pathology and dermatopathology were more knowledgeable about the technique (Supplementary Table 1).

**Table 1:** Distribution of answers to questions regarding background knowledge about AI.

**Question**	**Yes**	**No**				**log(OR) for age -group (95% CI)**	***P-value***	**Holm corrected *P*-value**	**log(OR) for sex (95% CI) [Ref. Female]**	***P*-value**	**Holm corrected *P*-value**
AI is a topic that has become of interest for the pathology community. Were you already aware of this topic in pathology?	585 (81.5%)	133 (18.5%)				0.10 (−0.08, 0.28)	0.28	1	−0.03 (-0.42, 0.37)	0.89	1
Have you read any medical publications regarding AI within dermatopathology?	181 (25.2%)	537 (74.8%)				0.12 (−0.03, 0.27)	0.12	1	0.29 (−0.06, 0.63)	0.11	1
Have you used AI as a diagnostic aid in real life within pathology?	160 (22.3%)	558 (77.7%)				0.09 (−0.07, 0.25)	0.29	1	−0.18 (−0.55, 0.19)	0.35	1
Have you used AI as a diagnostic aid in real life within dermatopathology?	79 (11.0%)	639 (89.0%)				0.18 (−0.03, 0.38)	0.087	1	−0.07 (−0.56, 0.42)	0.78	1
	**I have never heard about it**	**I have heard about it, but not more**	**Basic knowledge**	**Good knowledge**	**Excellent knowledge**	**Score increase per age interval (95% CI)**	***P-value***	**Holm corrected** ***P*****-value**	**Sex;** **Score difference** **(95% CI) [Ref. Female]**	***P***	**Holm corrected** ***P*****-value**
Which degree of knowledge would you say you have when it comes to AI within pathology?	25 (3.5%)	275 (38.3%)	283 (39.4%)	111 (15.5%)	24 (3.3%)	0.06 (0.01, 0.12)	**0.034**	1	0.20 (0.07, 0.33)	**0.003**	0.12

*For the questions with dichotomous answers, log-odds ratios, 95% CI and P-values for the logistic regression model (no=0 and yes=1) containing both sex and age group (20-24, 25-34, 35-44, 45-54, 55-64, 65-74, and ≥75 years) are included. The age groups are used as numeric values in the regression model, i.e., numbers ranging from 1 to 7. For the final question with five possible answers, the answer was transformed to a numeric score (1–5) and a linear regression model with both sex and age group was used with the coefficients, 95% CI and P-values included in the table. AI, artificial intelligence; CI, confidence interval; OR, odds ratio. Bold values indicates the singificant values P < 0.05*.

Responders with ≤ 10 years of experience within pathology (339 of 423, 80.1%) were equally aware of the AI development as responders with >10 years of experience (237 of 285, 83.2%) (*P* = 0.33, *P*_*adj*_ = 1). In the tech-savvy group, 99 of 376 responders (26.3%) reported either good or excellent knowledge about AI within pathology compared to 36 of 342 non-tech-savvy responders (10.5%) (*P* < 0.0001, *P*_*adj*_ < 0.0001).

When responders were asked about previous exposure to AI as a topic in general (Q4-Q7), 71.0, 74.9, 53.5, and 65.5% had heard about AI from the media, social media, lectures and friends, respectively. Social media and friends were more common sources for younger age groups. Other media was a more common source among men compared to females (Table 2).

**Table 2:** Distribution of answers to questions regarding sources about AI applications.

**Other applications we use in daily life already use AI (e.g., speech recognition, spam filters, recommendation algorithms). Have you been made aware of the use of AI in such applications?**	**Yes**	**No**	**log(OR) for age -group (95% CI)**	***P*-value**	**Holm corrected *P*-value**	**log(OR) for sex (95% CI) [Ref. Female]**	***P*-value**	**Holm corrected *P*-value**
From the media	510 (71.0%)	208 (29.0%)	0.07 (−0.08, 0.23)	0.37	1	0.53 (0.18, 0.88)	**0.003**	0.14
From social media	538 (74.9%)	180 (25.1%)	−0.30 (−0.46, −0.15)	**0.0001**	**0.004**	0.27 (−0.10, 0.63)	0.15	1
From lectures	384 (53.5%)	334 (46.5%)	0.11 (−0.03, 0.25)	0.12	1	0.17 (−0.14, 0.48)	0.27	1
From friends	470 (65.5%)	248 (34.5%)	−0.16 (−0.30, −0.02)	**0.029**	1	0.37 (0.04, 0.70)	**0.029**	1

*A logistic regression model (no = 0 and yes = 1) containing both sex and age group (20-24, 25-34, 35-44, 45-54, 55-64, 65-74, and ≥75 years) was used. The age groups are used as numeric values in the regression model, i.e., numbers ranging from 1 to 7. Log-odds ratios, 95% CI and P-values for the coefficients are included. AI, artificial intelligence; CI, confidence interval; OR, odds ratio. Bold values indicates the singificant values P < 0.05*.

**Figure 2:**
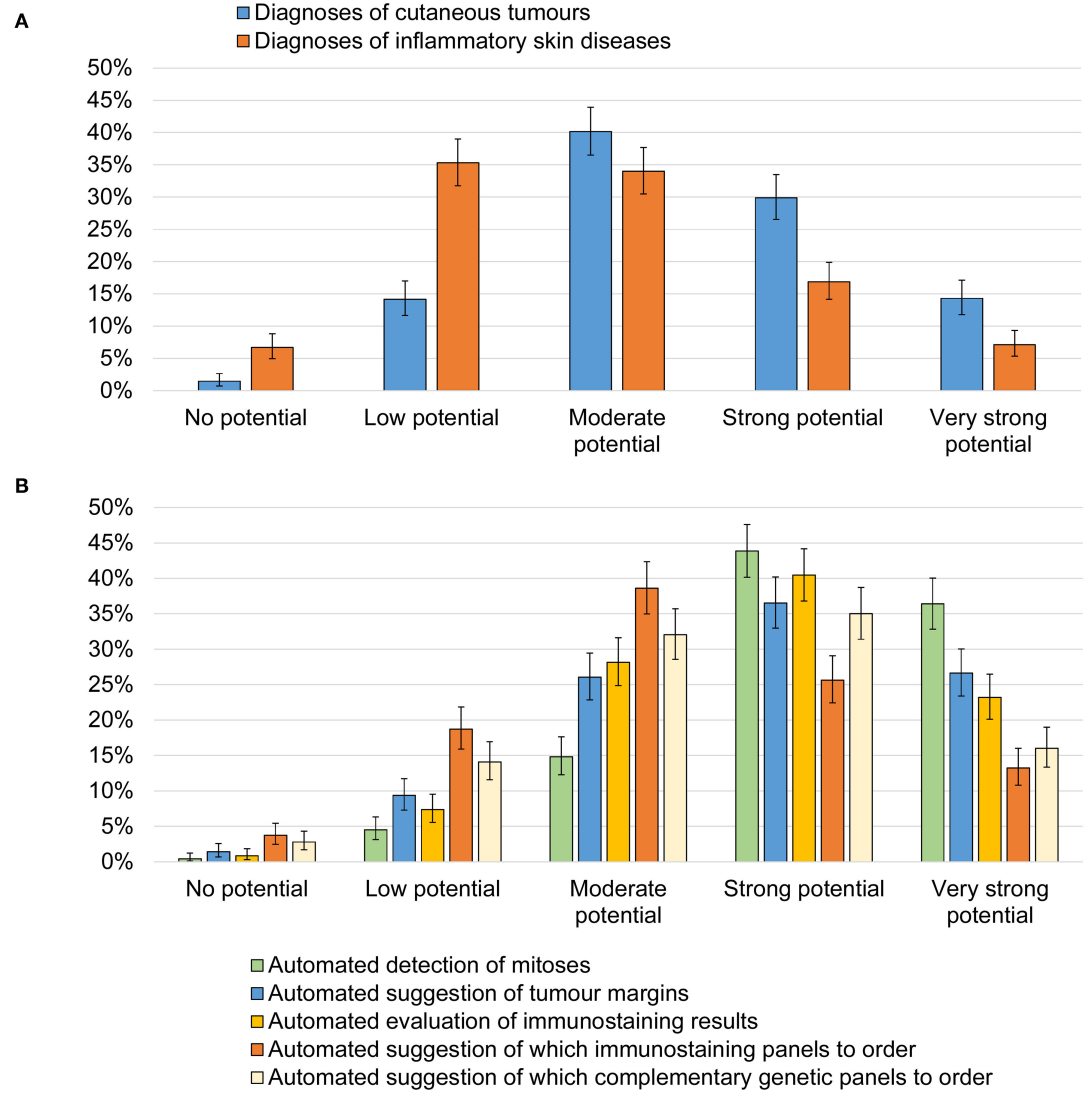
Potential seen for AI within dermatopathology. Regardless of whether you have thought about this before, which potential do you personally see for AI for dermatopathology images regarding each of the following. **(A)** Potential seen for automated suggestion of diagnoses of cutaneous tumors and inflammatory skin diseases. **(B)** Potential seen for specific tasks including; automated detection of mitoses; automated suggestion of tumor margins; automated evaluation of immunostaining results; automated suggestion of which immunostaining panels to order; automated suggestion of which complementary genetic panels to order.

The aggregated results from Q8-Q14 about seven applications for AI within dermatopathology are summarized in Figure 2. In terms of classification of diagnoses, 42.6% (*n* = 306) saw strong or very strong potential for automated suggestion of skin tumor diagnoses. The corresponding figure for inflammatory skin diseases was 23.0% (*n* = 165) (*P* < 0.0001, *P*_*adj*_ < 0.0001). For specific applications, the highest potential was considered for automated detection of mitosis (79.2%), automated suggestion of tumor margins (62.1%) and immunostaining evaluation (62.7%). The potential for automated suggestion of immunostaining (37.6%) and genetic panels (48.3%) were lower.

Results for feelings and attitudes toward AI (Q15-Q25) are presented in Table 3. Age did not impact the overall attitudes toward AI. Males displayed more excitement and less fear about the use of AI within dermatopathology as well as within medicine in general. The level of fear toward a development with an increased use of AI within dermatopathology did not differ between dermatopathologists working mainly at a university hospital compared to those who mainly worked in another setting (16.7 vs. 15.8%, *P* = 0.76, *P*_*adj*_ = 1). Among 280 responders with known access to WSI, 14.6% (*n* = 41) expressed fear toward a development with an increase use of AI within dermatopathology, which did not differ significantly to expressed fears among 74 of 421 responders without such access (17.6%) (*P* = 0.35, *P*_*adj*_ = 1). Furthermore, tech-savviness did not influence expressed levels of fear (data not shown). The attitudes toward AI among those with and without access to WSI did not differ, whereas those with previous use of the technique in dermatopathology had generally more positive attitudes (Supplementary Table 2).

**Table 3:** Distribution of answers to questions regarding attitudes and feelings about AI.

**Question**	**Strongly disagree**	**Disagree**	**Neither agree nor disagree**	**Agree**	**Strongly agree**	**I don't know**	**Score increase per age interval (95% CI)**	***P*-value**	**Holm corrected *P*-value**	**Sex; Score difference (95% CI) [Ref. Female]**	***P*-value**	**Holm corrected *P*-value**
AI will revolutionize Medicine in general.	3 (0.4%)	59 (8.2%)	122 (17.0%)	405 (56.4%)	129 (18.0%)	0 (0.0%)	0.02 (−0.04, 0.071)	0.56	1	0.21 (0.08, 0.33)	**0.001**	**0.046**
AI will revolutionize dermatopathology.	7 (1.0%)	64 (8.9%)	190 (26.5%)	322 (44.8%)	113 (15.7%)	22 (3.1%)	0.02 (−0.04, 0.08)	0.52	1	0.16 (0.02, 0.30)	**0.023**	0.80
AI will revolutionize dermatopathology more than other subfields within pathology.	22 (3.1%)	217 (30.2%)	304 (42.3%)	96 (13.4%)	37 (5.2%)	42 (5.8%)	−0.01 (−0.07, 0.05)	0.79	1	−0.02 (−0.16, 0.12)	0.74	1
In the foreseeable future all physicians will be replaced by AI.	250 (34.8%)	342 (47.6%)	70 (9.7%)	26 (3.6%)	17 (2.4%)	13 (1.8%)	0.01 (−0.06, 0.07)	0.84	1	−0.01 (−0.15, 0.13)	0.91	1
The human pathologist will be replaced by AI in the foreseeable future.	248 (34.5%)	337 (46.9%)	66 (9.2%)	30 (4.2%)	14 (1.9%)	23 (3.2%)	0.03 (−0.03, 0.09)	0.28	1	0.09 (−0.05, 0.22)	0.23	1
A development with an increased use of AI in dermatopathology frightens me.	78 (10.9%)	320 (44.6%)	204 (28.4%)	93 (13.0%)	23 (3.2%)	0 (0.0%)	−0.01 (−0.08, 0.05)	0.65	1	−0.17 (−0.32, −0.03)	**0.022**	0.79
A development with an increased use of AI in dermatopathology makes dermatopathology more exciting to me.	12 (1.7%)	69 (9.6%)	210 (29.2%)	340 (47.4%)	87 (12.1%)	0 (0.0%)	−0.03 (−0.09, 0.03)	0.35	1	0.18 (0.05, 0.31)	**0.009**	0.34
A development with an increased use of AI makes medicine in general more exciting to me.	8 (1.1%)	60 (8.4%)	186 (25.9%)	371 (51.7%)	93 (13.0%)	0 (0.0%)	0.01 (−0.05, 0.07)	0.73	1	0.17 (0.05, 0.30)	**0.008**	0.33
AI will improve dermatopathology	6 (0.8%)	36 (5.0%)	126 (17.5%)	425 (59.2%)	94 (13.1%)	31 (4.3%)	0.01 (−0.04, 0.06)	0.64	1	0.20 (0.09, 0.32)	**0.001**	**0.034**
AI will improve medicine in general.	3 (0.4%)	20 (2.8%)	93 (13.0%)	464 (64.6%)	116 (16.2%)	22 (3.1%)	0.00 (−0.04, 0.05)	0.84	1	0.26 (0.15, 0.36)	**<0.0001**	**<0.0001**
AI should be part of medical training.	6 (0.8%)	19 (2.6%)	72 (10.0%)	441 (61.4%)	163 (22.7%)	17 (2.4%)	0.06 (0.01, 0.11)	**0.017**	0.62	0.10 (−0.01, 0.21)	0.081	1
I consider myself well-informed about the use of modern technology, especially computers.	3 (0.4%)	59 (8.2%)	122 (17.0%)	405 (56.4%)	129 (18.0%)	0 (0.0%)	−0.03 (−0.09, 0.03)	0.31	1	0.18 (0.05, 0.30)	**0.0064**	0.26

*The five possible answers were transformed to a numeric score (1–5) used as the dependent variable and a linear regression model with both sex and age group as predictors was used with the coefficients, 95% CI and P-values included in the table. The age groups are used as numeric values in the regression model, i.e., numbers ranging from 1 to 7. All “I don't know” answers were excluded from the regression model. AI, artificial intelligence; CI, confidence interval. Bold values indicates the singificant values P < 0.05*.

Only 6.0% (43 of 718) of the responders agreed or strongly agreed that the human pathologist will be replaced by AI in the foreseeable future. Among tech-savvy responders, the corresponding figure was 7.7% (29 of 376, *P* = 0.086, *P*_*adj*_ = 1). For the entire group, 72.3% agreed or strongly agreed that AI will improve dermatopathology and 84.1% thought that AI should be a part of medical training.

## Discussion

Our results indicate a generally positive attitude toward AI within dermatopathology among surveyed pathologists. The majority of responders have not yet used AI tools as a diagnostic aid within dermatopathology, underlining that such tools are still under development and a broad implementation to a large extent still is pending.

Age did not influence the results and, while males generally expressed more excitement and less fear compared to female responders, the differences were small in absolute terms. Nonetheless, some of the differences between the genders, might be due to systematic gaps in confidence levels between men and women (19, 20). The majority of the responders were not afraid of being replaced by AI and felt that AI will improve dermatopathology making it more exciting. Only 4% of the responders mainly worked with digital pathology which could explain the low usage of AI tools. Interestingly, only 25% of the responders had read a medical publication regarding AI within dermatopathology which may reflect that studies in this field are still rare.

Narrow tasks such as automated detection of mitoses and tumor margins were seen to have higher potential compared to automated diagnosis of both cutaneous tumors and inflammatory skin disorders. This is not surprising since several studies have shown AI's potential in detecting mitoses while evidence in diagnostic use is still rare (21, 22). In regards to AI-driven automated diagnostics, its usefulness for tumors was considered more likely than for dermatoses. In fact, a few studies have shown the potential of AI in differentiating skin tumors in WSI (12, 23). AI has even outperformed pathologists in the classification of melanoma (24). Melanocytic lesions in specific have proven particularly difficult for dermatopathologists, where significant levels of inter- and intra-observer variability have been reported (25). ML-based solutions likely hold strong promise in this domain.

The responders reported a high potential for automated suggestion of tumor margins. Such benefits were observed in a recent study in which fluorescence lifetime imaging and ML were used to visualize tumor margins in excised breast specimens showing high specificity and sensitivity (26). In a previous survey about general pathologists' attitudes toward AI, 75% of the pathologists showed interest or excitement about AI as a diagnostic tool and the vast majority felt it could increase diagnostic efficiency (27). This is in line with the findings in our survey.

As the fields of dermatology and dermatopathology are getting ready for a tremendous transition, we believe inventories of attitudes toward AI are important since it will clarify the potential in specific domains and hopefully unify the community to try to work together. Future ML tools will probably make use of several sources of information simultaneously and parallel systems might provide an even more accurate output. Recently, online dermatopathology forums have collected large amounts of digital cases. This online educational resource could potentially be used to train convolutional neural networks. Ideally, these tools should be available through a web-browser and should be freely available to all users. We believe that pathologists must engage in this development in order to take a leading position in AI. The positive attitudes presented here could certainly help promote this development. Nonetheless, this survey also illustrates that the majority (61.0%) of responders don't yet have access to WSI. A broad implementation of WSI is most likely a prerequisite for an increased use of AI in dermatopathology.

When interpreting our results, it is imperative to remember that the majority of responders received the survey invitation via their social media interest in dermatopathology. The possibility of a selection bias exists and physicians with positive attitudes may have been more likely to have completed the survey. Moreover, AI is a new technique and different responders might have varying ideas of what it includes and represents, particularly since the processes between input and output by the algorithms are not usually clearly defined, and the methods and variable combinations may differ from our own decision-making.

Setting up an online link rather than solely inviting pathologists from a predetermined mailing list also makes it difficult to obtain an exact survey response rate. We acknowledge that attitudes toward new technologies including AI can be fluid and have a tendency to change over time. Therefore, it will also be interesting to reassess attitudes toward AI in follow-up investigations. Moreover, we recognize the inherent difficulties in analyzing and merging the results of attitudes. Undoubtedly, the same question and answer options can represent different meanings to different responders who also have different baseline knowledge about the technology. Finally, ethical and legal aspects, which may vary between countries, were not addressed in our survey.

## Conclusion

In summary, our results demonstrate an overall optimistic attitude toward AI in dermatopathology among surveyed physicians. Nonetheless, our data also highlight the need for education about AI for pathologists. Surveyed pathologists predict greater potential for automated suggestion of skin tumor diagnoses than for inflammatory skin diseases. The greatest potential of AI in dermatopathology was predicted for automated detection of mitoses and tumor margins as well as for immunostaining evaluation.

## Data Availability Statement

The anonymized datasets generated in this survey are available on request to the corresponding author.

## Ethics Statement

Ethical review and approval was not required for the study on human participants in accordance with the local legislation and institutional requirements. Written informed consent for participation was not required for this study in accordance with the national legislation and the institutional requirements.

## Author Contributions

SP and JP contributed to study concept and design, supervised the project, and wrote the manuscript. SP carried out the implementation, data collection, analysis, and statistics with contributions from MG. MG provided biostatistical advice. PM, JG, JS, and NN contributed to design and distribution of the questionnaire. All authors critically reviewed and edited the questionnaire and the manuscript.

## Conflict of Interest

The authors declare that the research was conducted in the absence of any commercial or financial relationships that could be construed as a potential conflict of interest.

## Supplementary Material

Supplementary Material 1 Complete survey text.

Supplementary Material 2 Aggregated survey responses.

Supplementary Table 1 Distribution of answers to questions regarding background knowledge about AI. Proportions of answers to questions on the specific rows stratified according to access to WSI, previous use of AI in pathology and previous use of AI in dermatopathology. P-values are computed with Fisher's exact test.

Supplementary Table 2 Distribution of answers to questions regarding attitudes and feelings about AI. Proportions of answers to questions on the specific rows stratified according to access to WSI, previous use of AI in pathology and previous use of AI in dermatopathology. P-values are computed with Fisher's exact test.
