# Supplementary material for: Attitudes Toward Artificial Intelligence Within Dermatopathology: An International Online Survey
Source: Front Med (Lausanne). 2020 Oct 20;7:591952. doi: 10.3389/fmed.2020.591952 (PMC7606983; doi:10.3389/fmed.2020.591952)
Supplement: Supplementary Material 1 — Complete survey text. [file Data_Sheet_1.PDF]

Attitudes towards Artificial intelligence among pathologists working within dermatopathology

1. Introduction

March 15, 2020
Dear Colleague:

The Departments of Dermatology and Pathology at the University of Gothenburg are asking you for your valuable input. Your participation in this survey will provide us with important insight about pathologists' attitudes towards artificial intelligence (AI) in the field of dermatopathology.

All results will be statistically summarized and combined with those of your colleagues to provide a telling picture about attitudes towards AI in Dermatopathology. Upon completion, it is our intention to publish the results in a peer-reviewed open-access pathology journal. Your answers are important regardless of your level of experience and knowledge about AI.

The survey should only take 5 minutes, and your responses are completely anonymous.

You can only take the survey once. Questions marked with an asterisk (*) are required.

If you have any questions or comments regarding the survey, please feel free to contact principal investigator Sam Polesie at: sam.polesie@vgregion.se

Thank you in advance for taking the time to complete this survey.

Sincerely,
John Paoli, Assoc. Prof., co-investigator
Jerad Gardner, Assoc. Prof., co-investigator
Phillip H McKee, MD, FRCPath., co-investigator
Sam Polesie, PhD, principal investigator

2. Baseline question.

* 1. I work as a clinical pathologist and regularly analyze dermatopathology slides/images.

- ◯ Yes
- ◯ No

3. Part 1 of 5 **AI as a topic in Pathology**

* 2. AI is a topic that has become of interest for the pathology community. Were you already aware of this topic in pathology?

- ◯ Yes
- ◯ No

* 3. Which degree of knowledge would you say you have when it comes to AI within pathology?

- ◯ Excellent knowledge
- ◯ Good knowledge
- ◯ Basic knowledge
- ◯ I have heard about it, but not more
- ◯ I have never heard about it

4. Part 2 of 5 **Previous exposure from different sources to AI as a topic in general**

Other applications we use in daily life already use AI (e.g. speech recognition, spam filters, recommendation algorithms).
Have you been made aware of the use of AI in such applications?

* 4. From the media

- Yes
- No

* 5. From social media

- Yes
- No

* 6. From lectures

- Yes
- No

* 7. From friends

- Yes
- No

5. Part 3 of 5 **Applications for AI in dermatopathology**

Regardless of whether you have thought about this before, which potential do you personally see for AI for dermatopathology images regarding each of the following.

* 8. Automated suggestion of diagnoses of cutaneous tumours.

- Very strong potential
- Strong potential
- Moderate potential
- Low potential
- No potential
- I don't know

* 9. Automated suggestion of diagnoses of inflammatory skin diseases.

- Very strong potential
- Strong potential
- Moderate potential
- Low potential
- No potential
- I don't know

* 10. Automated detection of mitoses.

- Very strong potential
- Strong potential
- Moderate potential
- Low potential
- No potential
- I don't know

* 11. Automated suggestion of tumour margins.

- Very strong potential
- Strong potential
- Moderate potential
- Low potential
- No potential
- I don't know

* 12. Automated suggestion of which immunostaining panels to order.

- Very strong potential
- Strong potential
- Moderate potential
- Low potential
- No potential
- I don't know

* 13. Automated evaluation of immunostaining results.

- Very strong potential
- Strong potential
- Moderate potential
- Low potential
- No potential
- I don't know

* 14. Automated suggestion of which complementary genetic panels to order.

- Very strong potential
- Strong potential
- Moderate potential
- Low potential
- No potential
- I don't know

6. Part 4 of 5 **Feelings and attitudes towards AI**

In your personal opinion, to what extent do you agree to the following statements?

* 15. AI will revolutionize <u>medicine in general</u>.

- Strongly agree
- Agree
- Neither agree nor disagree
- Disagree
- Strongly disagree
- I don't know

* 16. AI will revolutionize <u>dermatopathology</u>.

- Strongly agree
- Agree
- Neither agree nor disagree
- Disagree
- Strongly disagree
- I don't know

* 17. AI will revolutionize dermatopathology <u>more than</u> other subfields within pathology.

- Strongly agree
- Agree
- Neither agree nor disagree
- Disagree
- Strongly disagree
- I don't know

* 18. In the foreseeable future all physicians will be replaced by AI.

- Strongly agree
- Agree
- Neither agree nor disagree
- Disagree
- Strongly disagree
- I don't know

* 19. The human pathologist will be replaced by AI in the foreseeable future.

- Strongly agree
- Agree
- Neither agree nor disagree
- Disagree
- Strongly disagree
- I don't know

* 20. A development with an increased use of AI in dermatopathology frightens me.

- Strongly agree
- Agree
- Neither agree nor disagree
- Disagree
- Strongly disagree

* 21. A development with an increased use of AI in dermatopathology makes dermatopathology more exciting to me.

- Strongly agree
- Agree
- Neither agree nor disagree
- Disagree
- Strongly disagree

* 22. A development with an increased use of AI makes medicine in general more exciting to me.

- Strongly agree
- Agree
- Neither agree nor disagree
- Disagree
- Strongly disagree

* 23. AI will improve dermatopathology

- Strongly agree
- Agree
- Neither agree nor disagree
- Disagree
- Strongly disagree
- I don't know

* 24. AI will improve medicine in general.

- Strongly agree
- Agree
- Neither agree nor disagree
- Disagree
- Strongly disagree
- I don't know

* 25. AI should be part of medical training.

- Strongly agree
- Agree
- Neither agree nor disagree
- Disagree
- Strongly disagree
- I don't know

7. Part 5 of 5 **Self-reported tech-savviness and demographics**

* 26. I consider myself well-informed about the use of modern technology, especially computers.

- Strongly agree
- Agree
- Neither agree nor disagree
- Disagree
- Strongly disagree

* 27. Would you consider yourself to be someone who enjoys technology?

- Yes
- No

* 28. Would you consider yourself to be tech-savvy? (i.e. knowing a lot about modern technology and how to use it)

- Yes
- No

* 29. Have you read any medical publications regarding AI within dermatopathology?

- Yes
- No

* 30. Have you used AI as a diagnostic aid in real life within pathology?

- Yes
- No

* 31. Have you used AI as a diagnostic aid in real life within dermatopathology?

- Yes
- No

* 32. I have access to whole slide imaging (digital access to pathology slides) in the pathology office where I work.

- Yes
- No
- I don't know

* 33. When I do routine diagnostic dermatopathology I mainly use:

- A microscope (glass slides)
- A computer (digitally scanned slides)

* 34. How many years have you been working within pathology?

- 0-5
- 6-10
- 11-15
- 16-20
- 21-25
- 26-30
- 31-35
- 36-40
- 40+

* 35. What is your age (years)?

* 36. What is your gender?

- Female
- Male
- Other

* 37. In what type of practice setting do you mainly work?

- Non-teaching hospital
- Community teaching hospital
- University hospital
- Private pathology lab
- Other (please specify)

* 38. Which best describes your current position?

- Resident
- Fellow
- Practicing pathologist
- Site group director
- Head of department
- Other (please specify)

* 39. In what country do you mainly work?

Thank you for your participation.
