# Supplementary material for: Attitudes Toward Artificial Intelligence Within Dermatopathology: An International Online Survey
Source: Front Med (Lausanne). 2020 Oct 20;7:591952. doi: 10.3389/fmed.2020.591952 (PMC7606983; doi:10.3389/fmed.2020.591952)
Supplement: Supplementary Material 2 — Aggregated survey responses. [file Data_Sheet_2.PDF]

Q1 I work as a clinical pathologist and regularly analyze dermatopathology slides/images.

Answered: 718 Skipped: 0

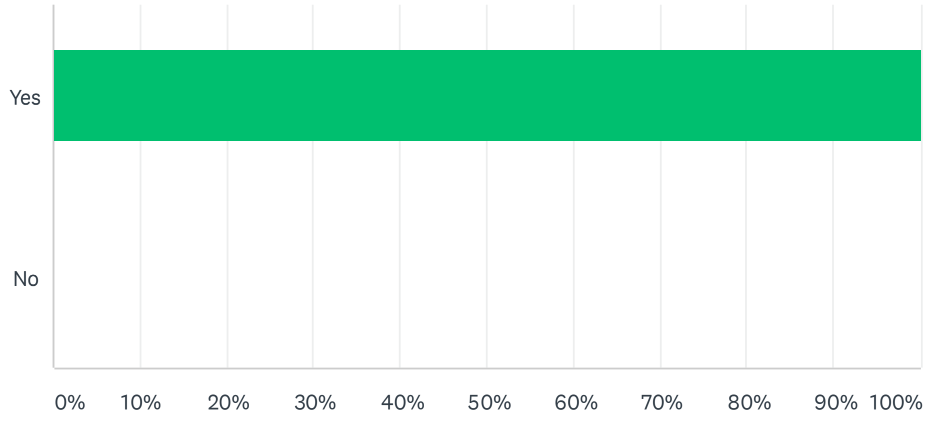

ANSWER CHOICES	RESPONSES	
Yes	100.00%	718
No	0.00%	0
TOTAL		718

Q2 AI is a topic that has become of interest for the pathology community. Were you already aware of this topic in pathology?

Answered: 718   Skipped: 0

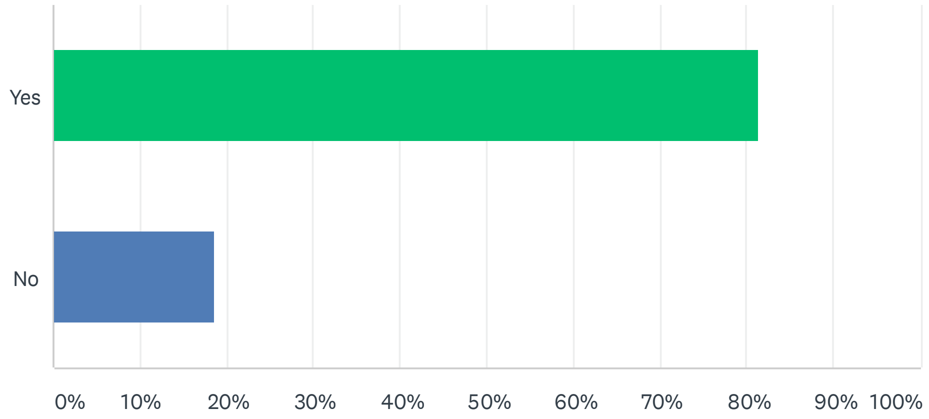

ANSWER CHOICES	RESPONSES	
Yes	81.48%	585
No	18.52%	133
TOTAL		718

Q3 Which degree of knowledge would you say you have when it comes to AI within pathology?

Answered: 718 Skipped: 0

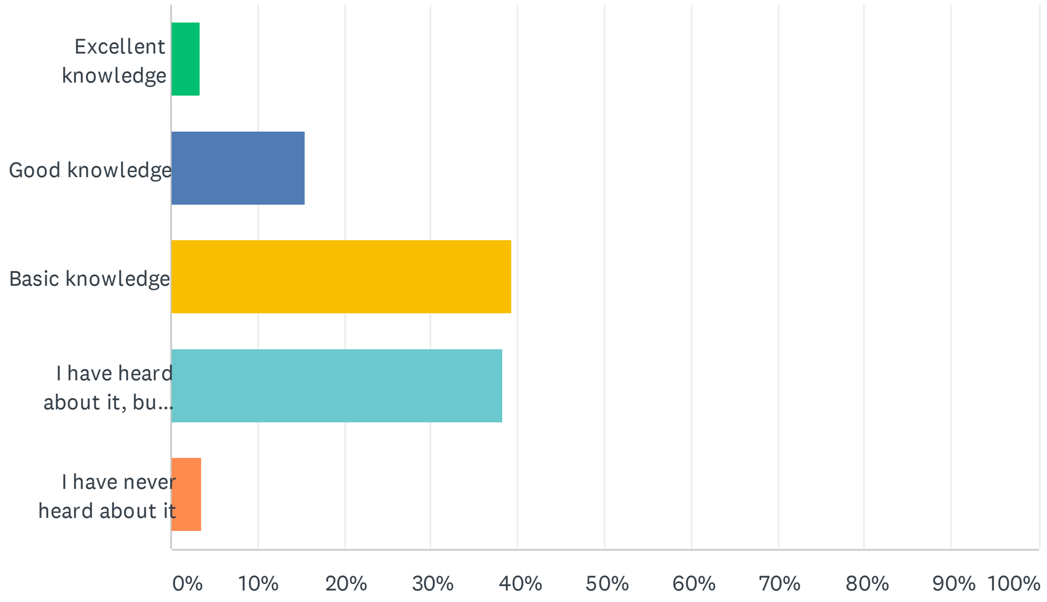

ANSWER CHOICES	RESPONSES	
Excellent knowledge	3.34%	24
Good knowledge	15.46%	111
Basic knowledge	39.42%	283
I have heard about it, but not more	38.30%	275
I have never heard about it	3.48%	25
TOTAL		718

Q4 From the media

Answered: 718 Skipped: 0

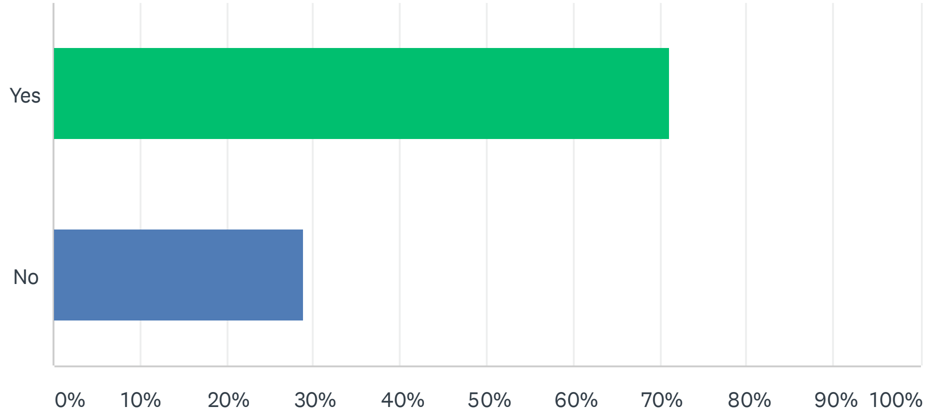

ANSWER CHOICES	RESPONSES	
Yes	71.03%	510
No	28.97%	208
TOTAL		718

Q5 From social media

Answered: 718 Skipped: 0

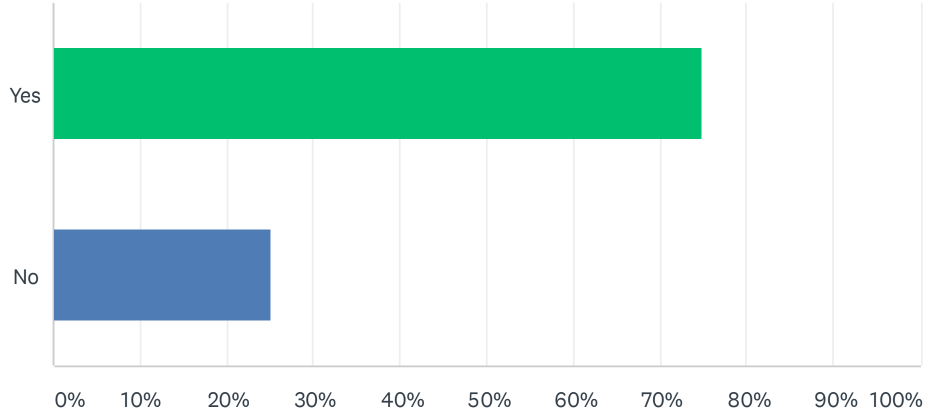

ANSWER CHOICES	RESPONSES	
Yes	74.93%	538
No	25.07%	180
TOTAL		718

Q6 From lectures

Answered: 718 Skipped: 0

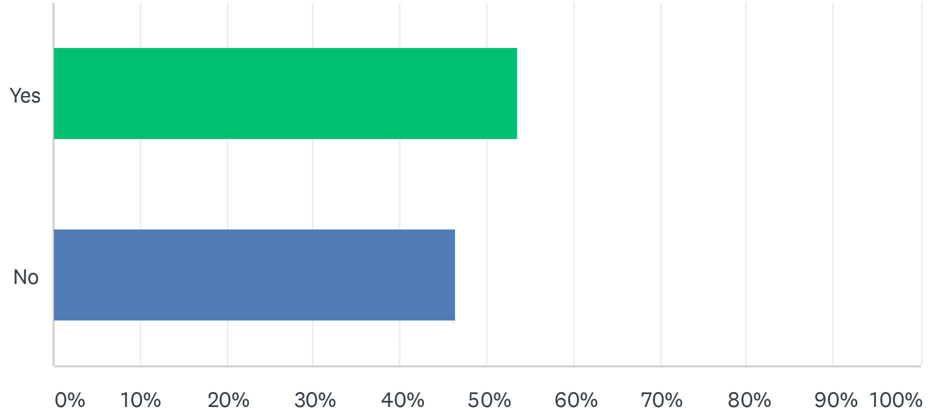

ANSWER CHOICES	RESPONSES	
Yes	53.48%	384
No	46.52%	334
TOTAL		718

Q7 From friends

Answered: 718   Skipped: 0

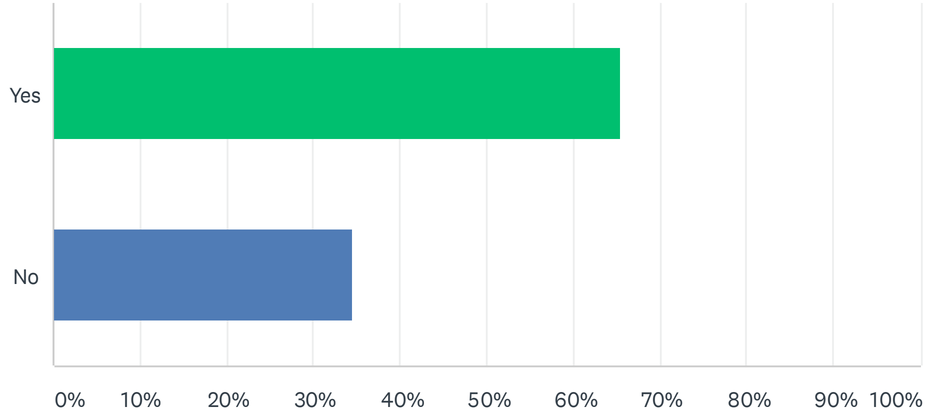

ANSWER CHOICES	RESPONSES	
Yes	65.46%	470
No	34.54%	248
TOTAL		718

Q8 Automated suggestion of diagnoses of cutaneous tumours.

Answered: 718 Skipped: 0

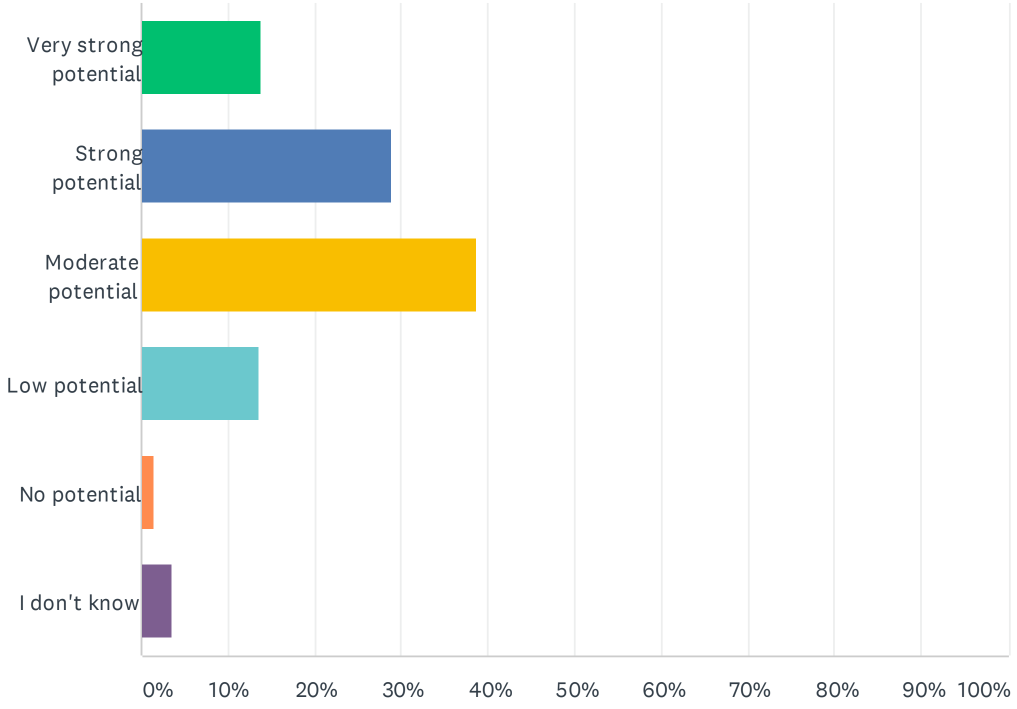

ANSWER CHOICES	RESPONSES	
Very strong potential	13.79%	99
Strong potential	28.83%	207
Moderate potential	38.72%	278
Low potential	13.65%	98
No potential	1.39%	10
I don't know	3.62%	26
TOTAL		718

Q9 Automated suggestion of diagnoses of inflammatory skin diseases.

Answered: 718 Skipped: 0

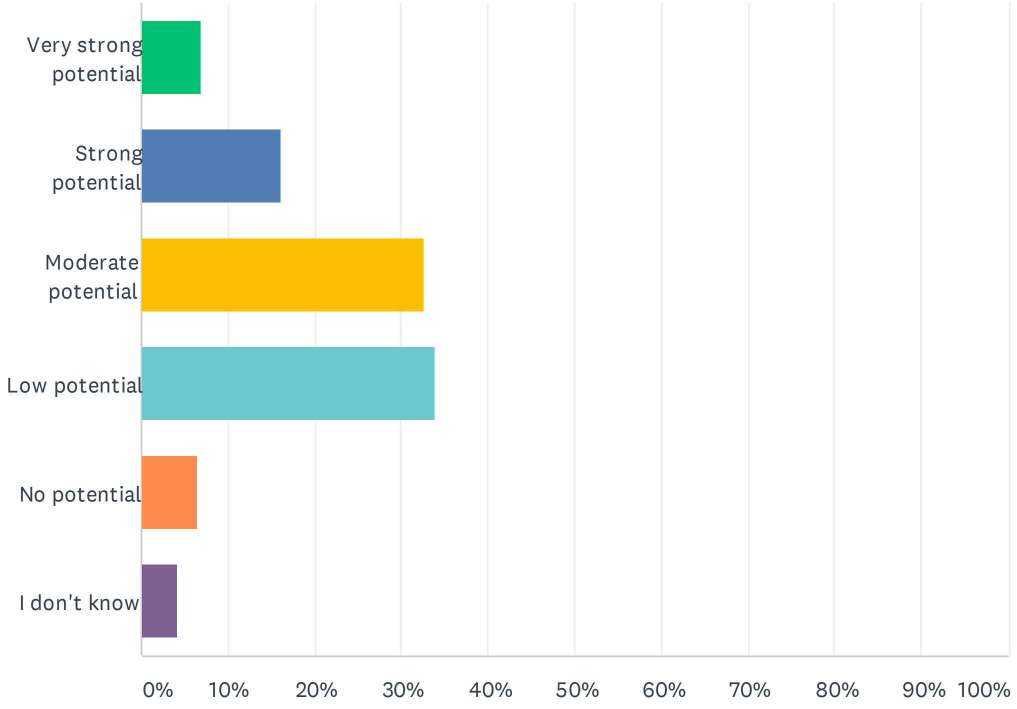

ANSWER CHOICES	RESPONSES	
Very strong potential	6.82%	49
Strong potential	16.16%	116
Moderate potential	32.59%	234
Low potential	33.84%	243
No potential	6.41%	46
I don't know	4.18%	30
TOTAL		718

Q10 Automated detection of mitoses.

Answered: 718 Skipped: 0

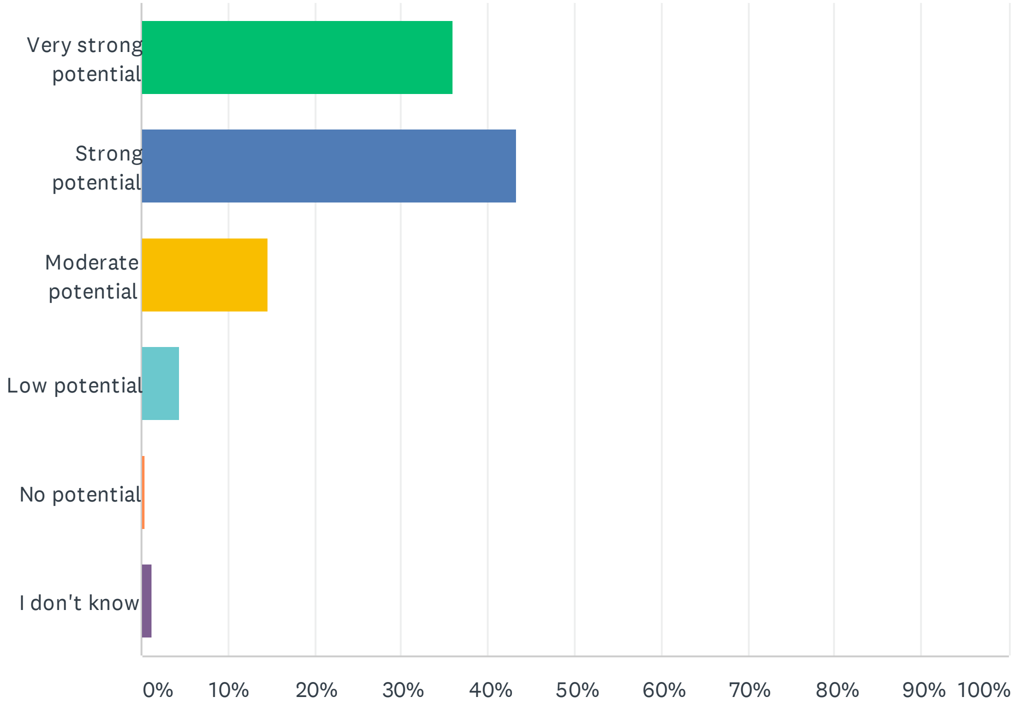

ANSWER CHOICES	RESPONSES	
Very strong potential	35.93%	258
Strong potential	43.31%	311
Moderate potential	14.62%	105
Low potential	4.46%	32
No potential	0.42%	3
I don't know	1.25%	9
TOTAL		718

Q11 Automated suggestion of tumour margins.

Answered: 718   Skipped: 0

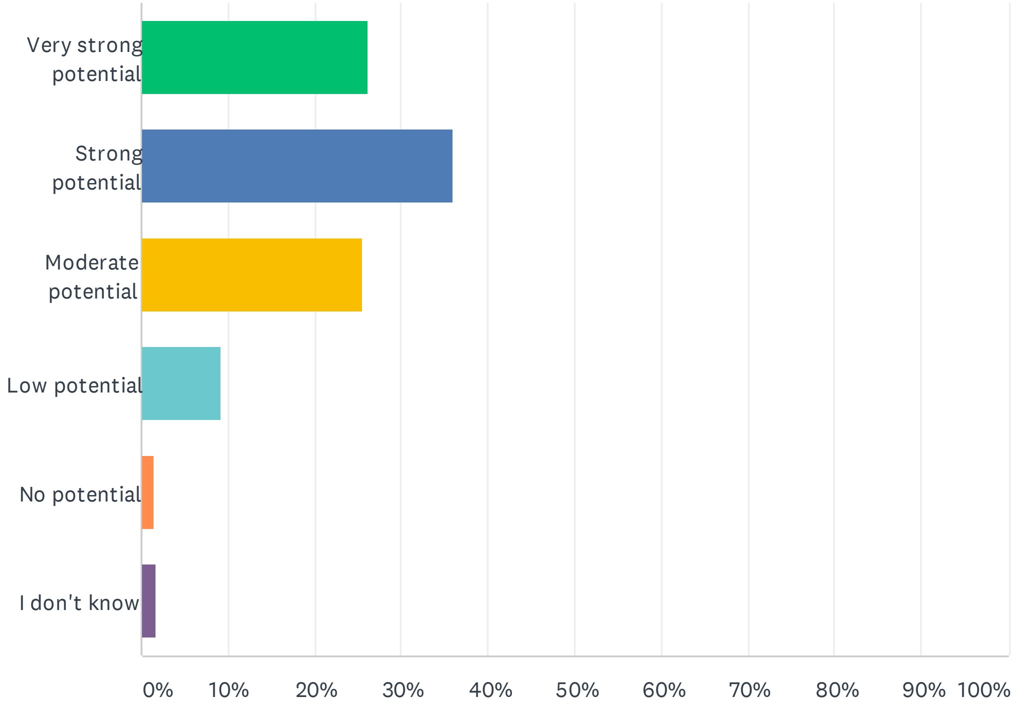

ANSWER CHOICES	RESPONSES	
Very strong potential	26.18%	188
Strong potential	35.93%	258
Moderate potential	25.63%	184
Low potential	9.19%	66
No potential	1.39%	10
I don't know	1.67%	12
TOTAL		718

Q12 Automated suggestion of which immunostaining panels to order.

Answered: 718 Skipped: 0

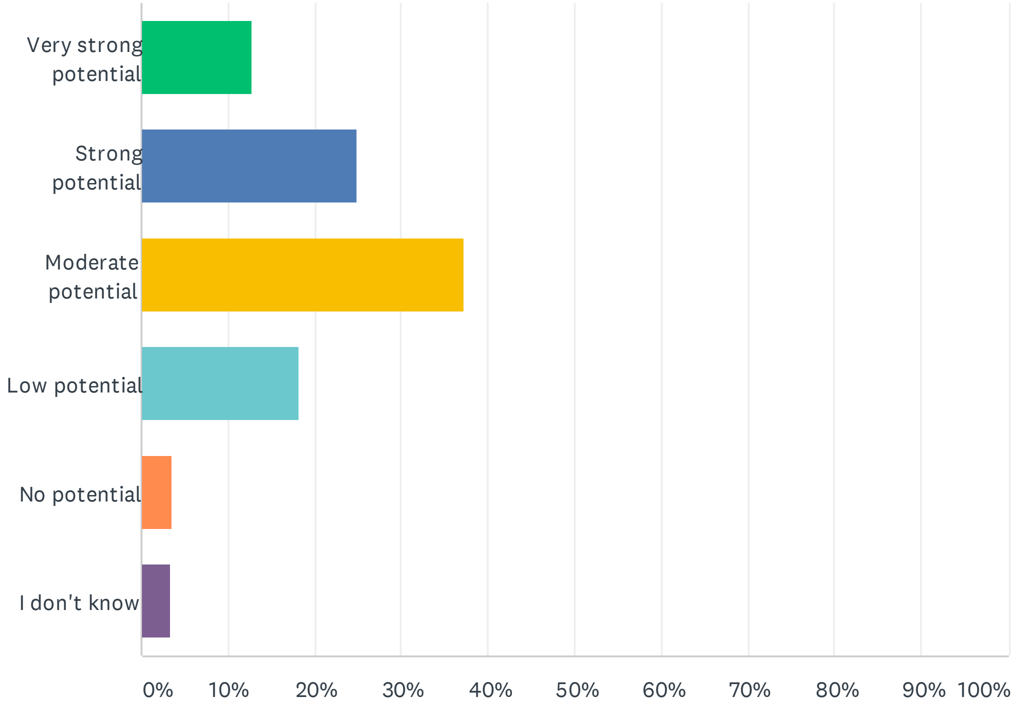

ANSWER CHOICES	RESPONSES	
Very strong potential	12.81%	92
Strong potential	24.79%	178
Moderate potential	37.33%	268
Low potential	18.11%	130
No potential	3.62%	26
I don't know	3.34%	24
TOTAL		718

Q13 Automated evaluation of immunostaining results.

Answered: 718   Skipped: 0

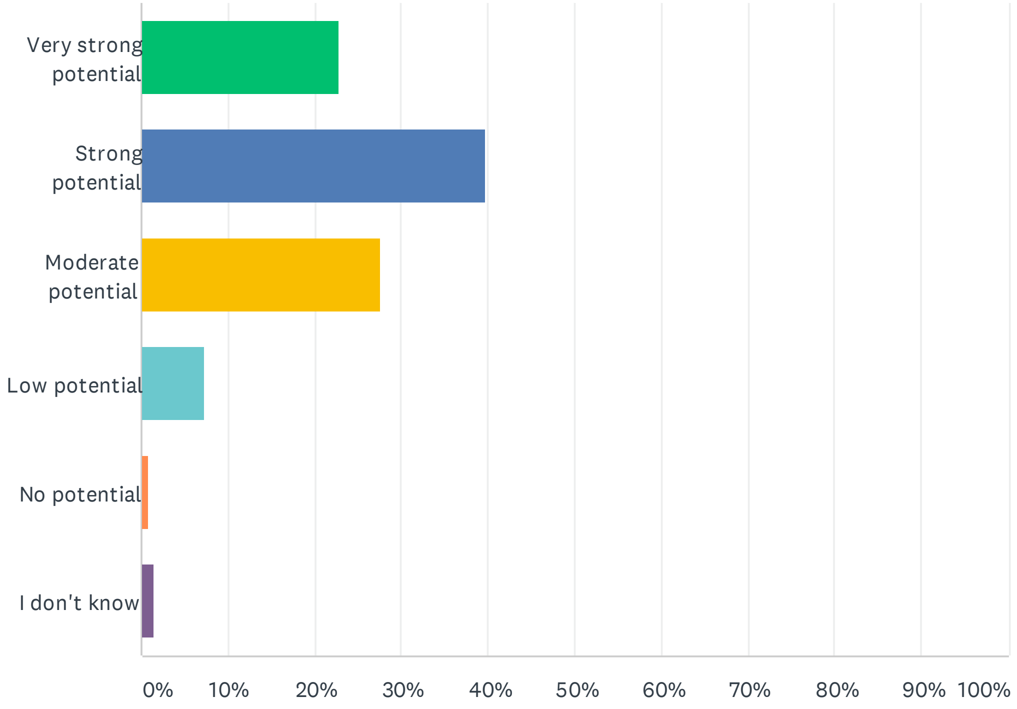

ANSWER CHOICES	RESPONSES	
Very strong potential	22.84%	164
Strong potential	39.83%	286
Moderate potential	27.72%	199
Low potential	7.24%	52
No potential	0.84%	6
I don't know	1.53%	11
TOTAL		718

Q14 Automated suggestion of which complementary genetic panels to order.

Answered: 718   Skipped: 0

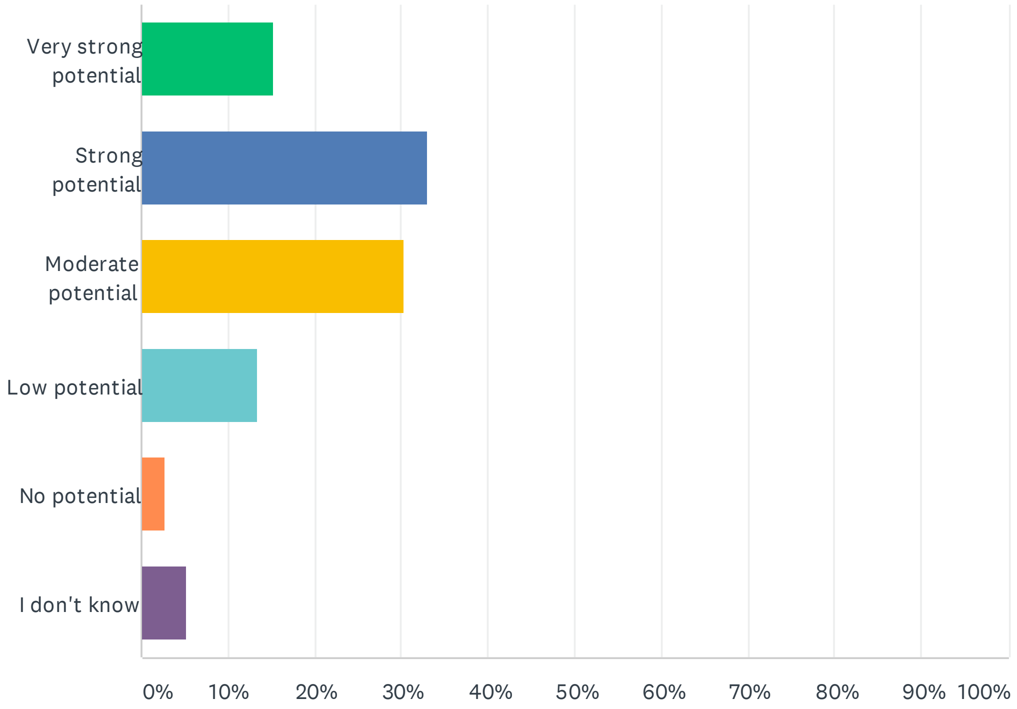

ANSWER CHOICES	RESPONSES	
Very strong potential	15.18%	109
Strong potential	33.15%	238
Moderate potential	30.36%	218
Low potential	13.37%	96
No potential	2.65%	19
I don't know	5.29%	38
TOTAL		718

Q15 AI will revolutionize medicine in general.

Answered: 718 Skipped: 0

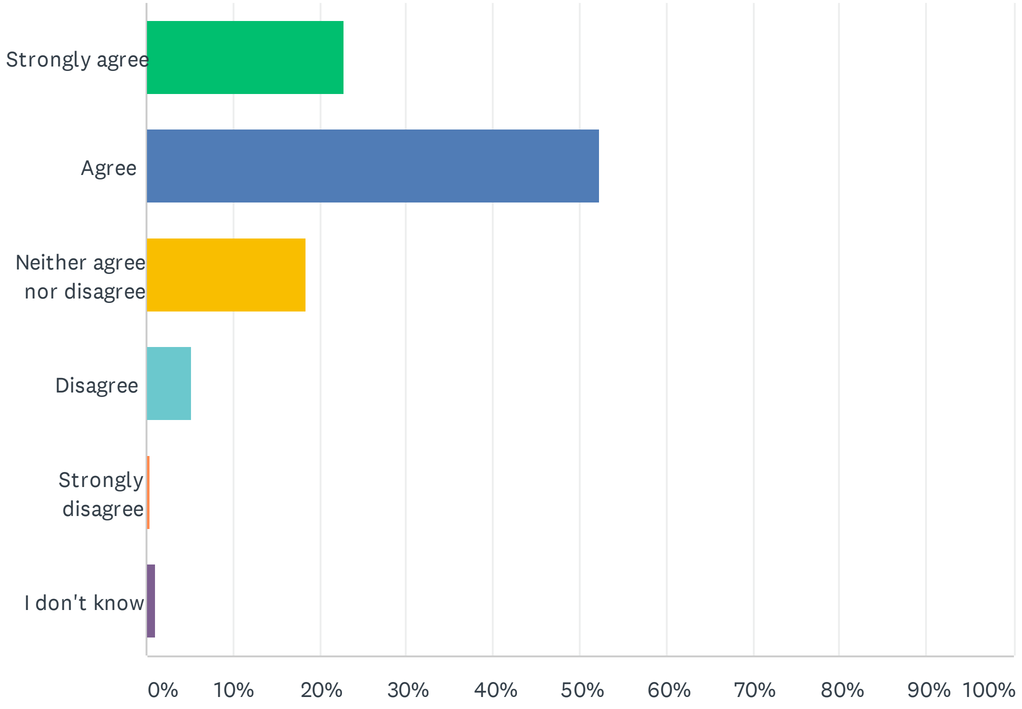

ANSWER CHOICES	RESPONSES	
Strongly agree	22.70%	163
Agree	52.23%	375
Neither agree nor disagree	18.38%	132
Disagree	5.15%	37
Strongly disagree	0.42%	3
I don't know	1.11%	8
TOTAL		718

Q16 AI will revolutionize dermatopathology.

Answered: 718 Skipped: 0

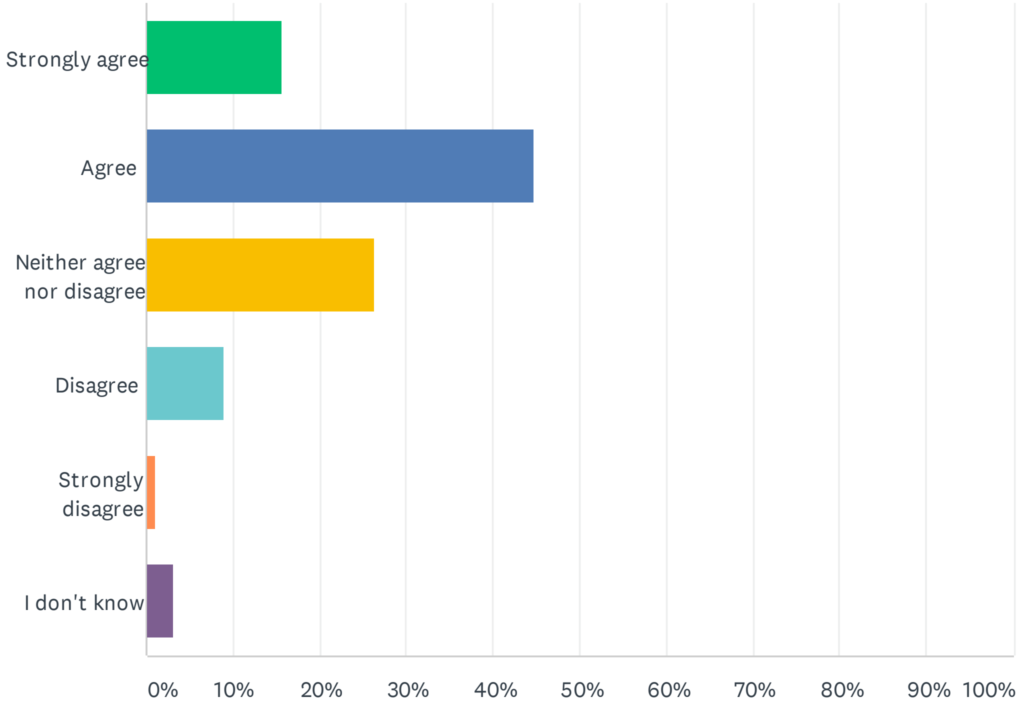

ANSWER CHOICES	RESPONSES	
Strongly agree	15.74%	113
Agree	44.85%	322
Neither agree nor disagree	26.46%	190
Disagree	8.91%	64
Strongly disagree	0.97%	7
I don't know	3.06%	22
TOTAL		718

Q17 AI will revolutionize dermatopathology more than other subfields within pathology.

Answered: 718 Skipped: 0

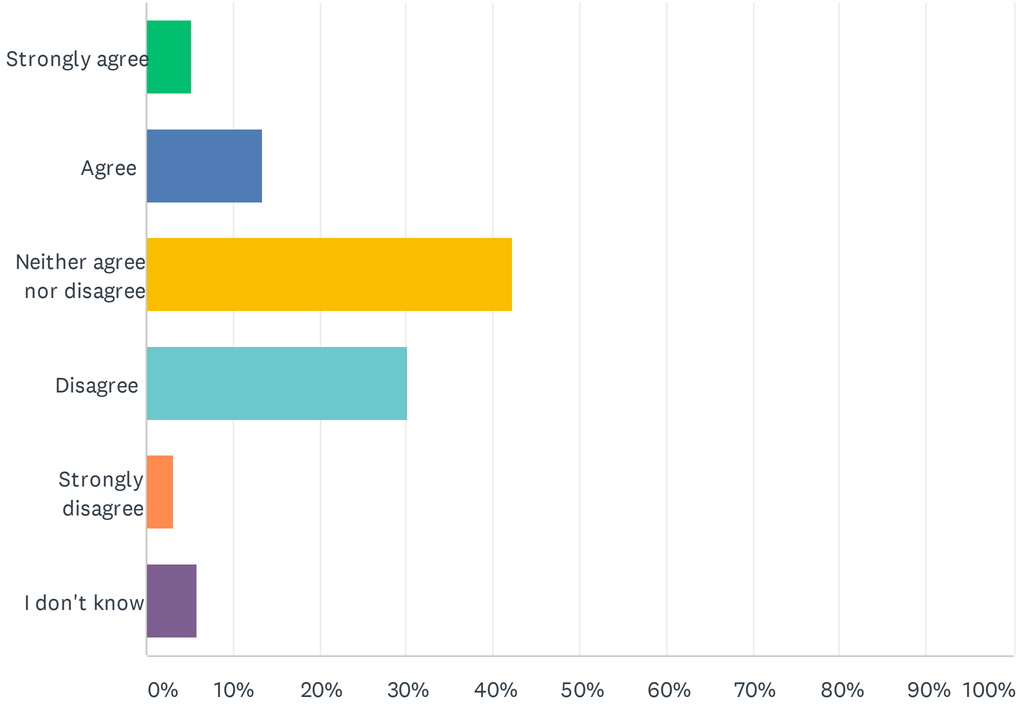

ANSWER CHOICES	RESPONSES	
Strongly agree	5.15%	37
Agree	13.37%	96
Neither agree nor disagree	42.34%	304
Disagree	30.22%	217
Strongly disagree	3.06%	22
I don't know	5.85%	42
TOTAL		718

Q18 In the foreseeable future all physicians will be replaced by AI.

Answered: 718 Skipped: 0

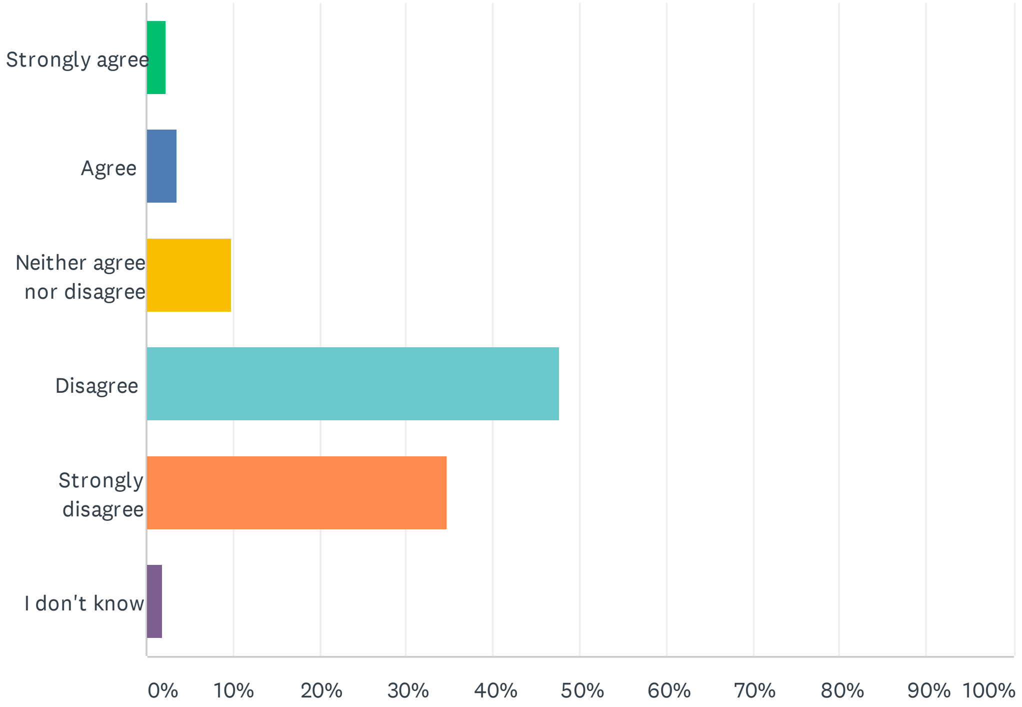

ANSWER CHOICES	RESPONSES	
Strongly agree	2.37%	17
Agree	3.62%	26
Neither agree nor disagree	9.75%	70
Disagree	47.63%	342
Strongly disagree	34.82%	250
I don't know	1.81%	13
TOTAL		718

Q19 The human pathologist will be replaced by AI in the foreseeable future.

Answered: 718 Skipped: 0

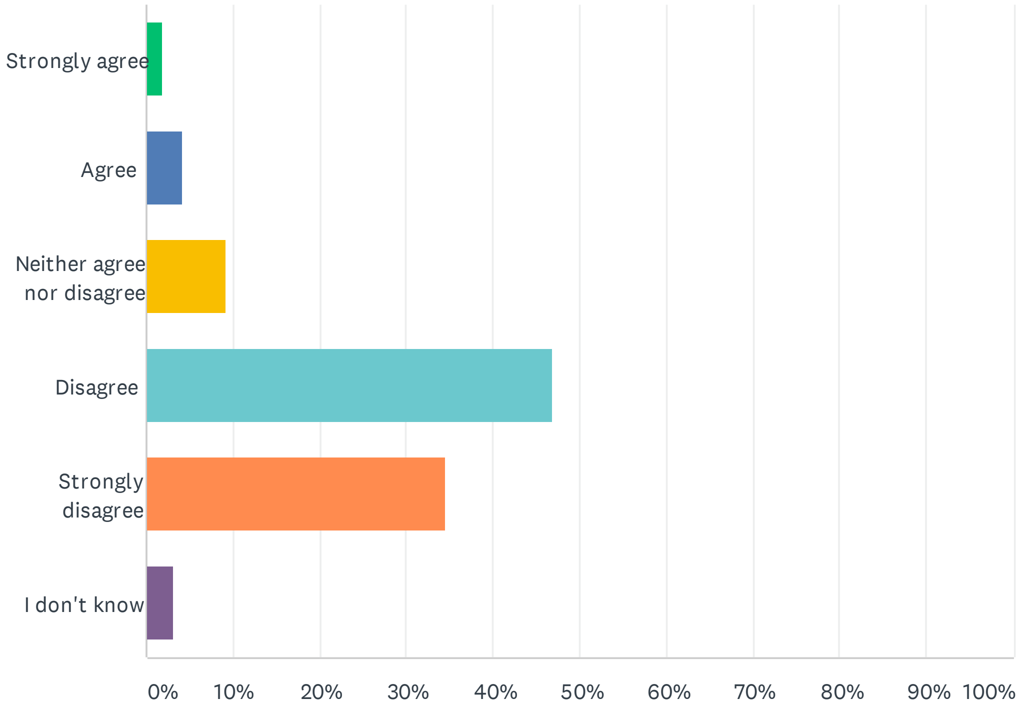

ANSWER CHOICES	RESPONSES	
Strongly agree	1.95%	14
Agree	4.18%	30
Neither agree nor disagree	9.19%	66
Disagree	46.94%	337
Strongly disagree	34.54%	248
I don't know	3.20%	23
TOTAL		718

Q20 A development with an increased use of AI in dermatopathology frightens me.

Answered: 718 Skipped: 0

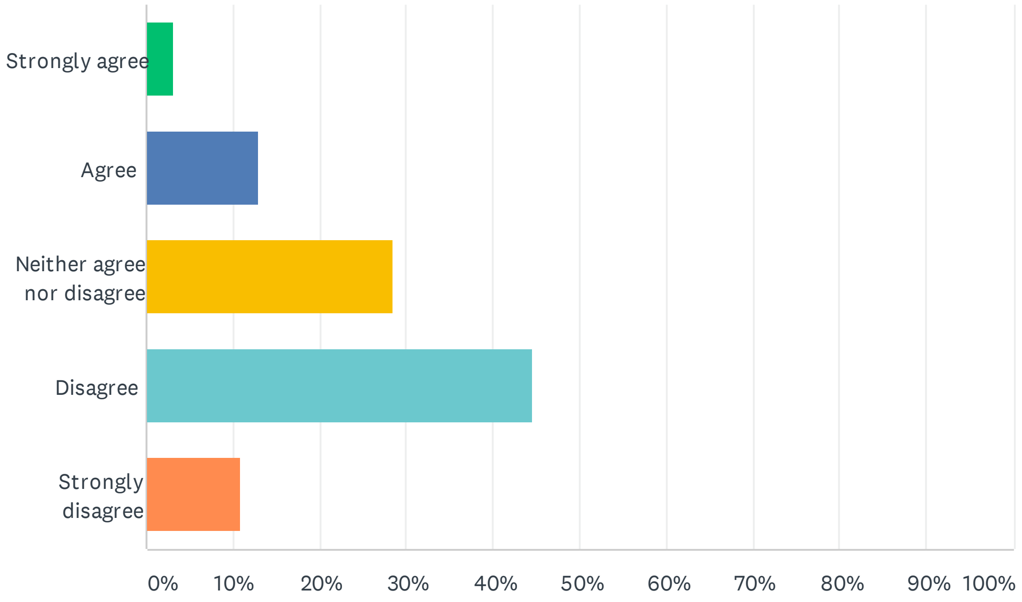

ANSWER CHOICES	RESPONSES	
Strongly agree	3.20%	23
Agree	12.95%	93
Neither agree nor disagree	28.41%	204
Disagree	44.57%	320
Strongly disagree	10.86%	78
TOTAL		718

Q21 A development with an increased use of AI in dermatopathology makes dermatopathology more exciting to me.

Answered: 718 Skipped: 0

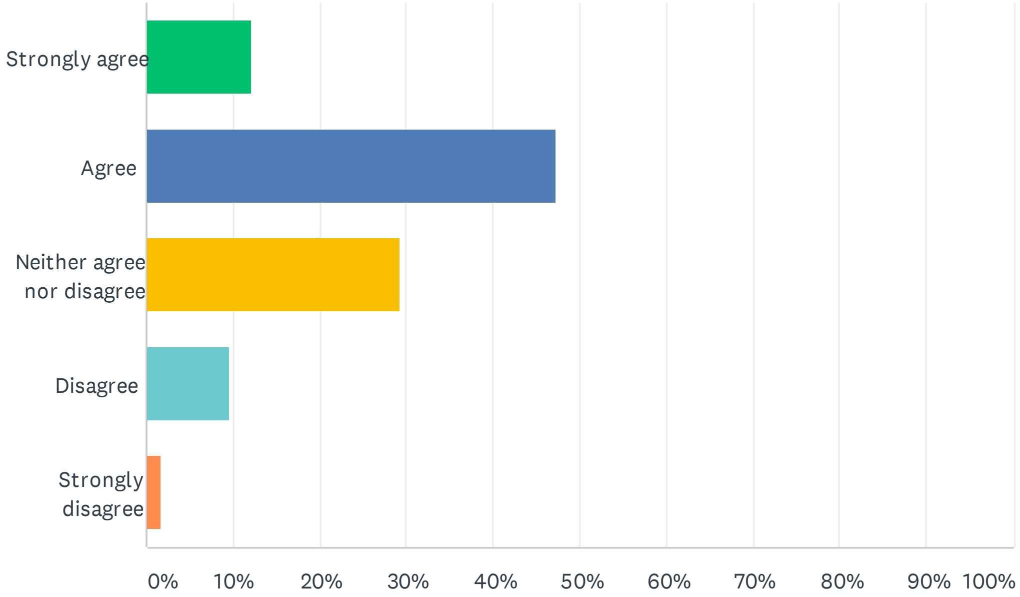

ANSWER CHOICES	RESPONSES	
Strongly agree	12.12%	87
Agree	47.35%	340
Neither agree nor disagree	29.25%	210
Disagree	9.61%	69
Strongly disagree	1.67%	12
TOTAL		718

Q22 A development with an increased use of AI makes medicine in general more exciting to me.

Answered: 718 Skipped: 0

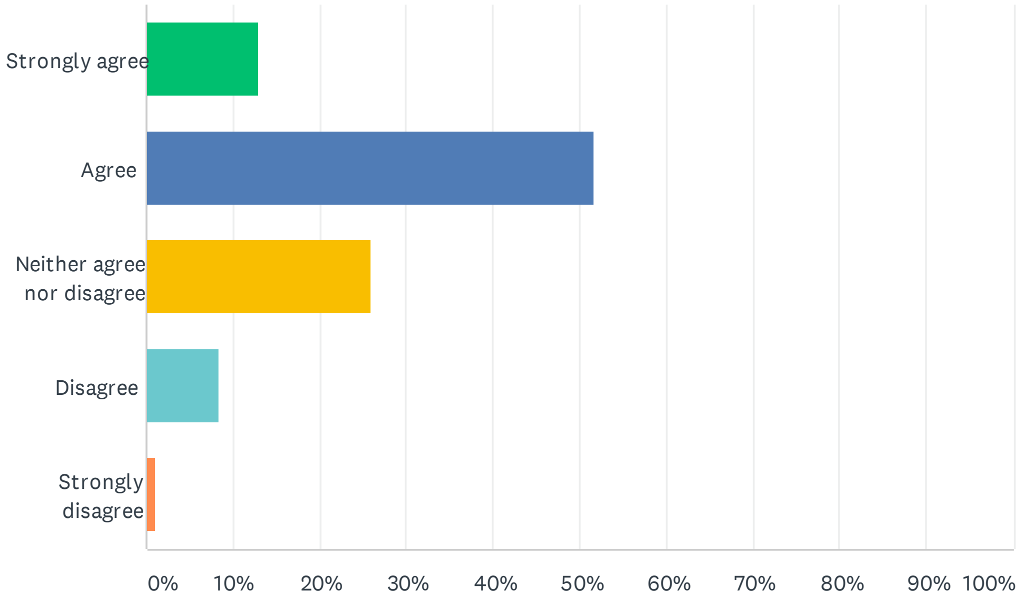

ANSWER CHOICES	RESPONSES	
Strongly agree	12.95%	93
Agree	51.67%	371
Neither agree nor disagree	25.91%	186
Disagree	8.36%	60
Strongly disagree	1.11%	8
TOTAL		718

Q23 AI will improve dermatopathology

Answered: 718 Skipped: 0

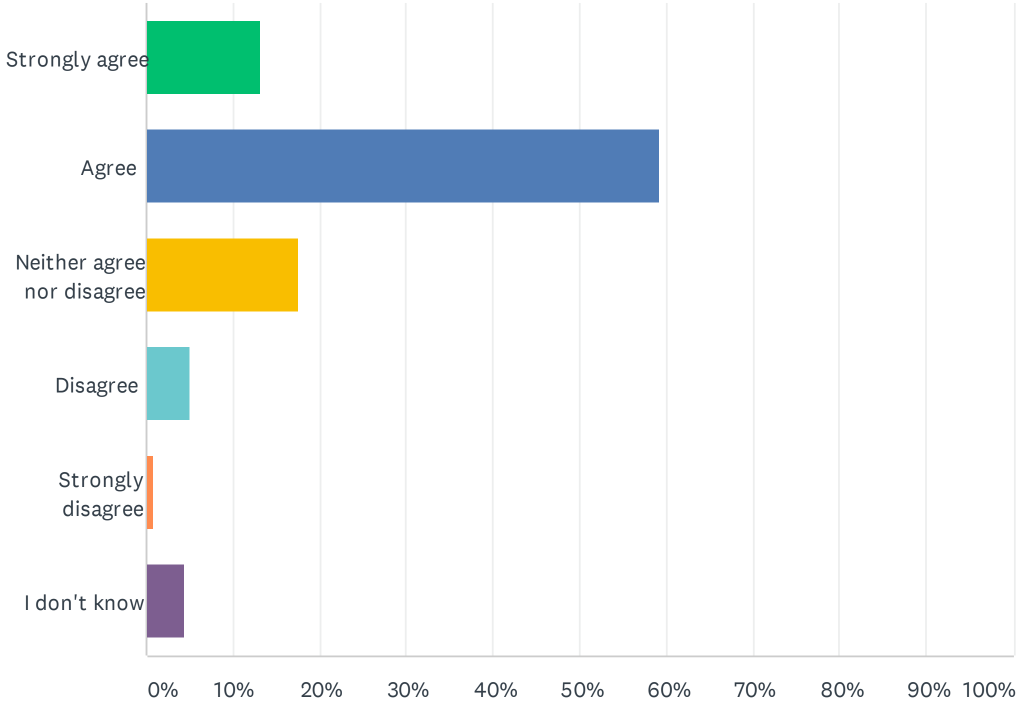

ANSWER CHOICES	RESPONSES	
Strongly agree	13.09%	94
Agree	59.19%	425
Neither agree nor disagree	17.55%	126
Disagree	5.01%	36
Strongly disagree	0.84%	6
I don't know	4.32%	31
TOTAL		718

Q24 AI will improve medicine in general.

Answered: 718   Skipped: 0

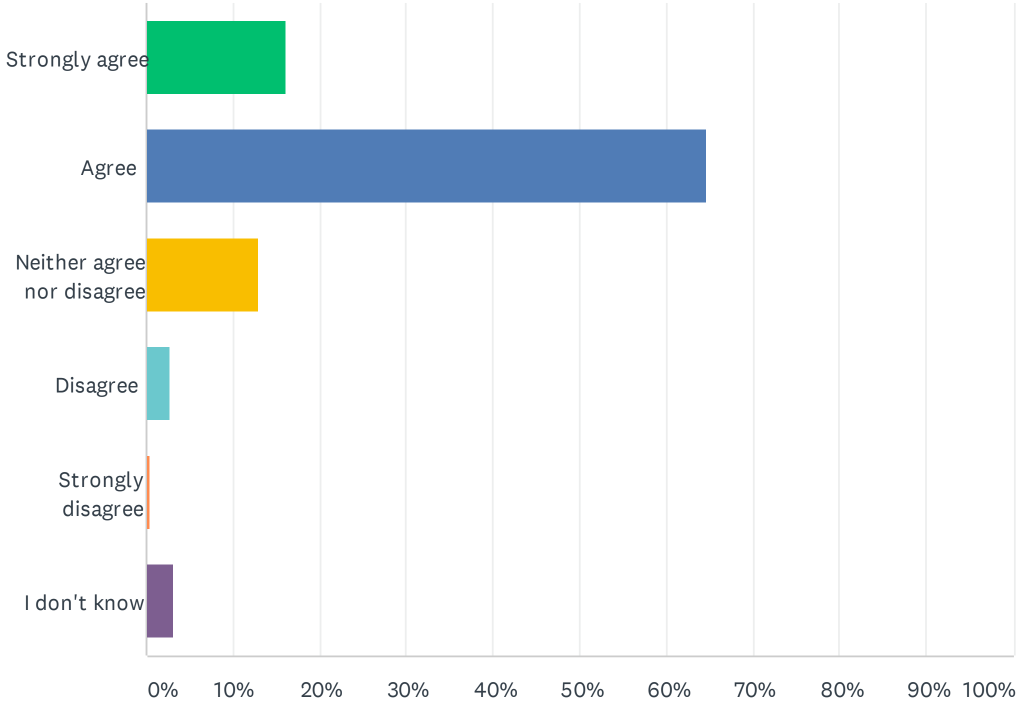

ANSWER CHOICES	RESPONSES	
Strongly agree	16.16%	116
Agree	64.62%	464
Neither agree nor disagree	12.95%	93
Disagree	2.79%	20
Strongly disagree	0.42%	3
I don't know	3.06%	22
TOTAL		718

Q25 AI should be part of medical training.

Answered: 718 Skipped: 0

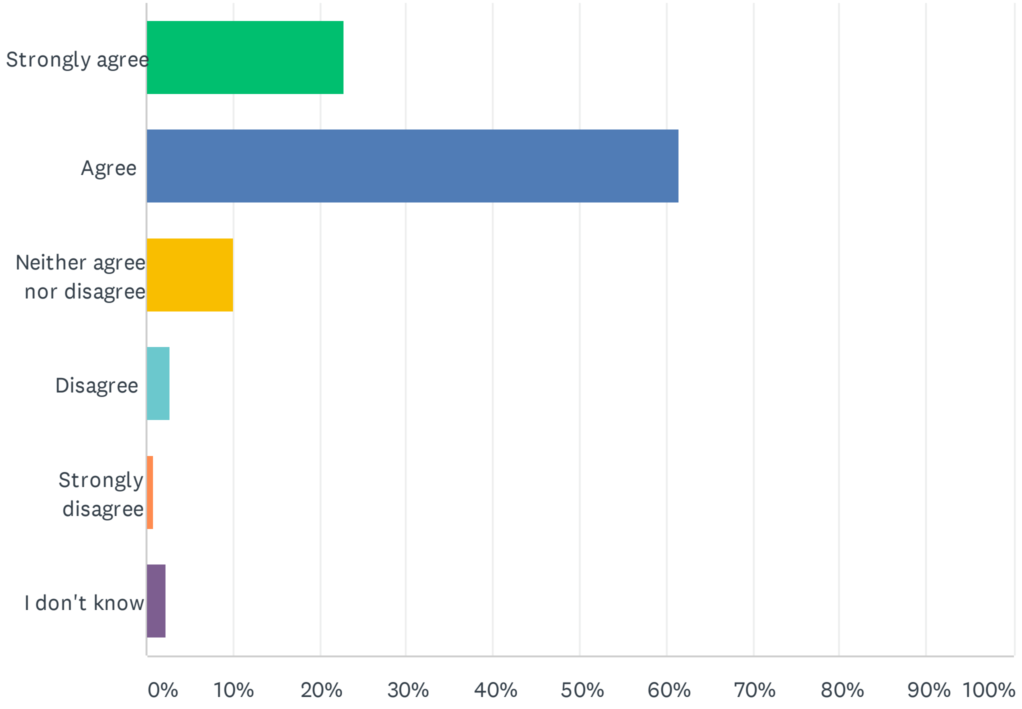

ANSWER CHOICES	RESPONSES	
Strongly agree	22.70%	163
Agree	61.42%	441
Neither agree nor disagree	10.03%	72
Disagree	2.65%	19
Strongly disagree	0.84%	6
I don't know	2.37%	17
TOTAL		718

Q26 I consider myself well-informed about the use of modern technology, especially computers.

Answered: 718 Skipped: 0

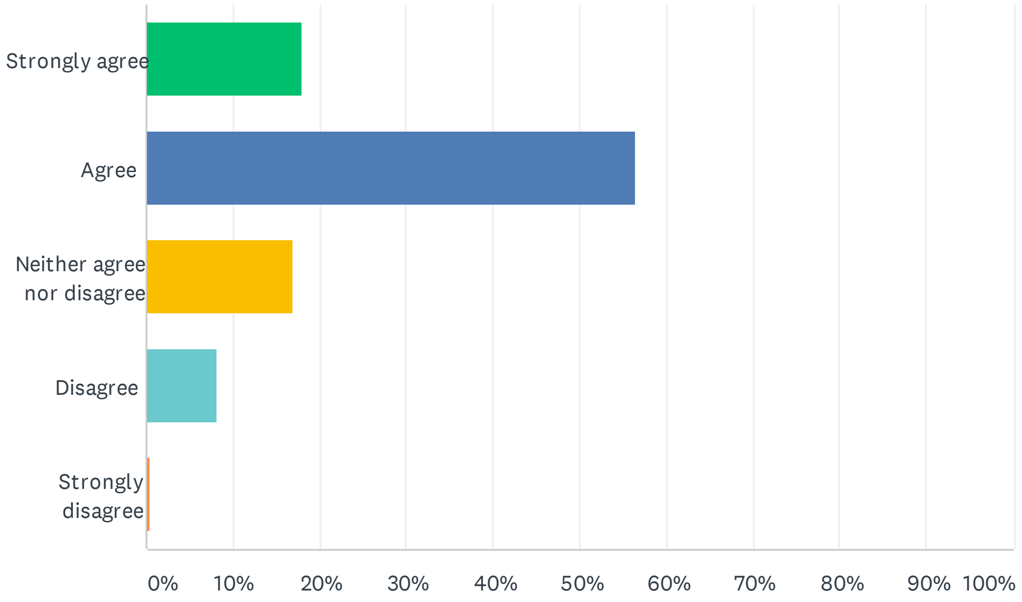

ANSWER CHOICES	RESPONSES	
Strongly agree	17.97%	129
Agree	56.41%	405
Neither agree nor disagree	16.99%	122
Disagree	8.22%	59
Strongly disagree	0.42%	3
TOTAL		718

Q27 Would you consider yourself to be someone who enjoys technology?

Answered: 718   Skipped: 0

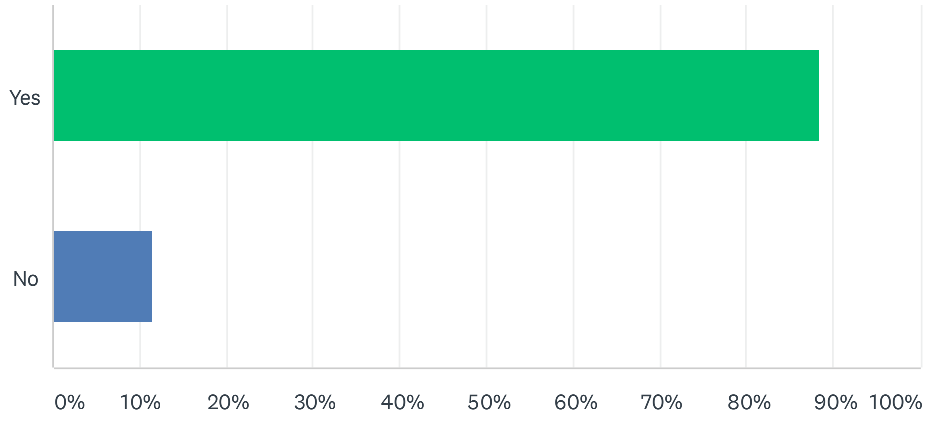

ANSWER CHOICES	RESPONSES	
Yes	88.58%	636
No	11.42%	82
TOTAL		718

Q28 Would you consider yourself to be tech-savvy? (i.e. knowing a lot about modern technology and how to use it)

Answered: 718 Skipped: 0

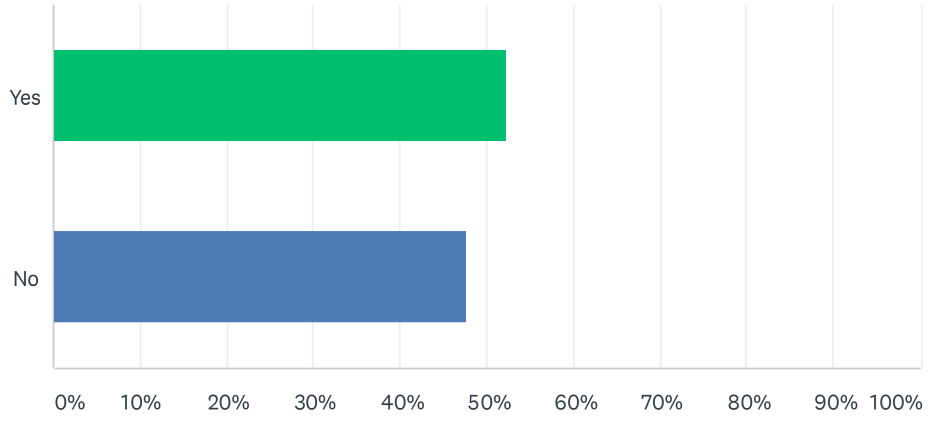

ANSWER CHOICES	RESPONSES	
Yes	52.37%	376
No	47.63%	342
TOTAL		718

Q29 Have you read any medical publications regarding AI within dermatopathology?

Answered: 718 Skipped: 0

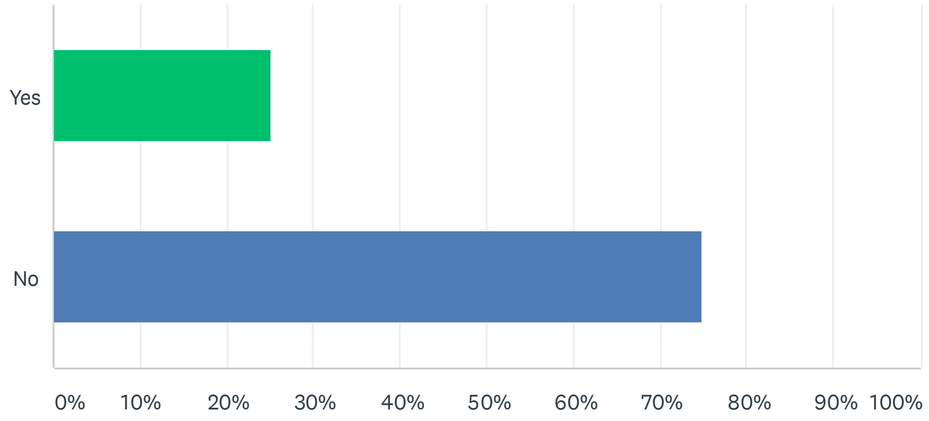

ANSWER CHOICES	RESPONSES	
Yes	25.21%	181
No	74.79%	537
TOTAL		718

Q30 Have you used AI as a diagnostic aid in real life within pathology?

Answered: 718 Skipped: 0

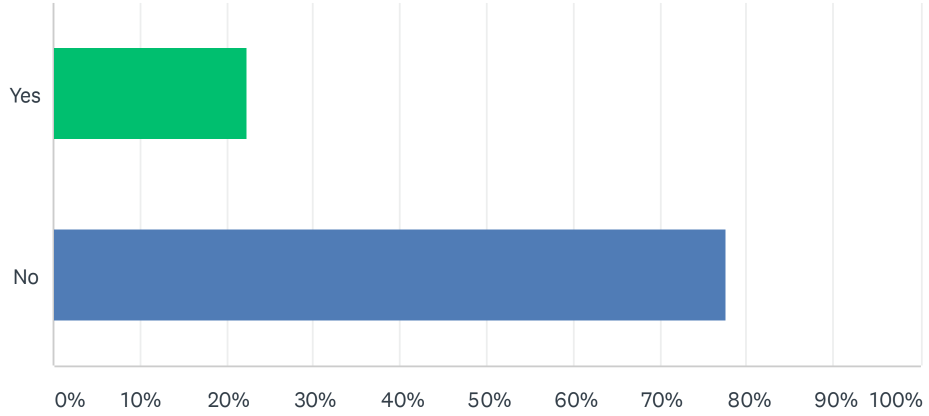

ANSWER CHOICES	RESPONSES	
Yes	22.28%	160
No	77.72%	558
TOTAL		718

Q31 Have you used AI as a diagnostic aid in real life within dermatopathology?

Answered: 718 Skipped: 0

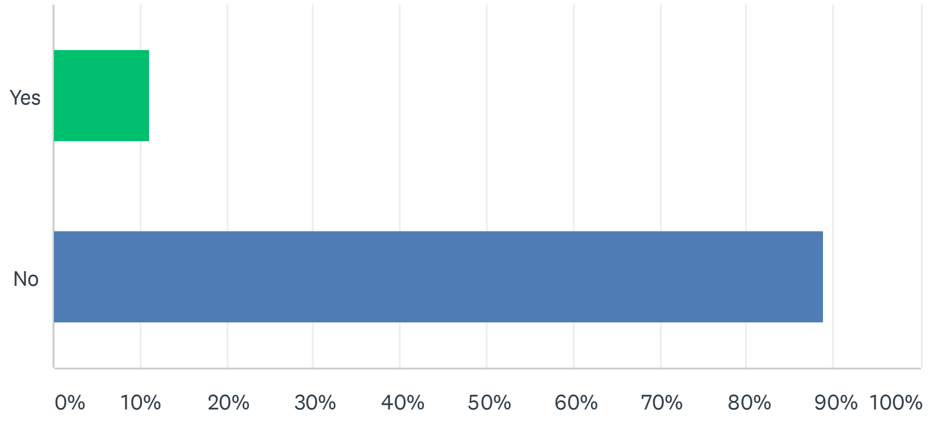

ANSWER CHOICES	RESPONSES	
Yes	11.00%	79
No	89.00%	639
TOTAL		718

Q32 I have access to whole slide imaging (digital access to pathology slides) in the pathology office where I work.

Answered: 718 Skipped: 0

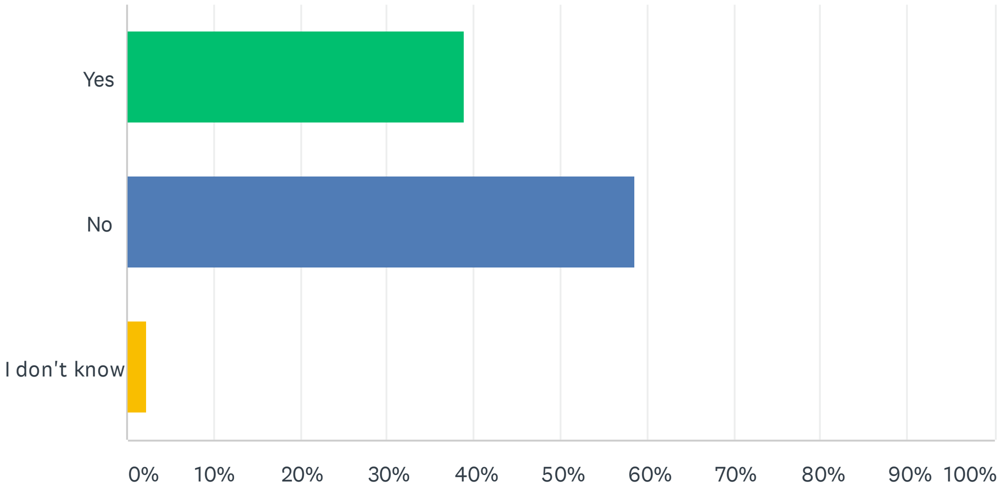

ANSWER CHOICES	RESPONSES	
Yes	39.00%	280
No	58.64%	421
I don't know	2.37%	17
TOTAL		718

Q33 When I do routine diagnostic dermatopathology I mainly use:

Answered: 718 Skipped: 0

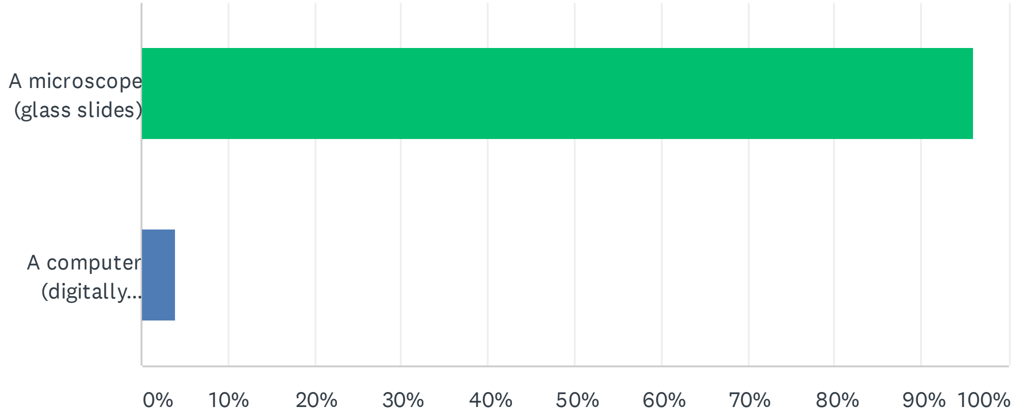

ANSWER CHOICES	RESPONSES	
A microscope (glass slides)	95.96%	689
A computer (digitally scanned slides)	4.04%	29
TOTAL		718

Q34 How many years have you been working within pathology?

Answered: 718 Skipped: 0

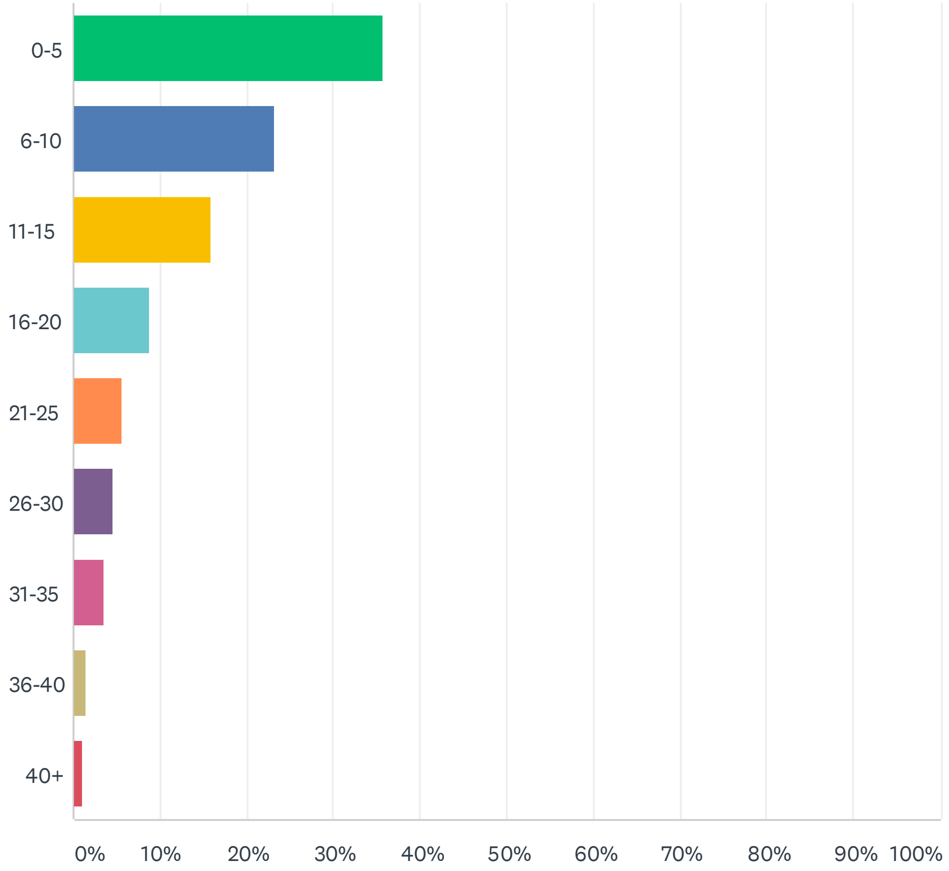

ANSWER CHOICES	RESPONSES	
0-5	35.79%	257
6-10	23.12%	166
11-15	15.88%	114
16-20	8.77%	63
21-25	5.71%	41
26-30	4.60%	33
31-35	3.62%	26
36-40	1.39%	10
40+	1.11%	8
TOTAL		718

Q35 What is your age (years)?

Answered: 718 Skipped: 0

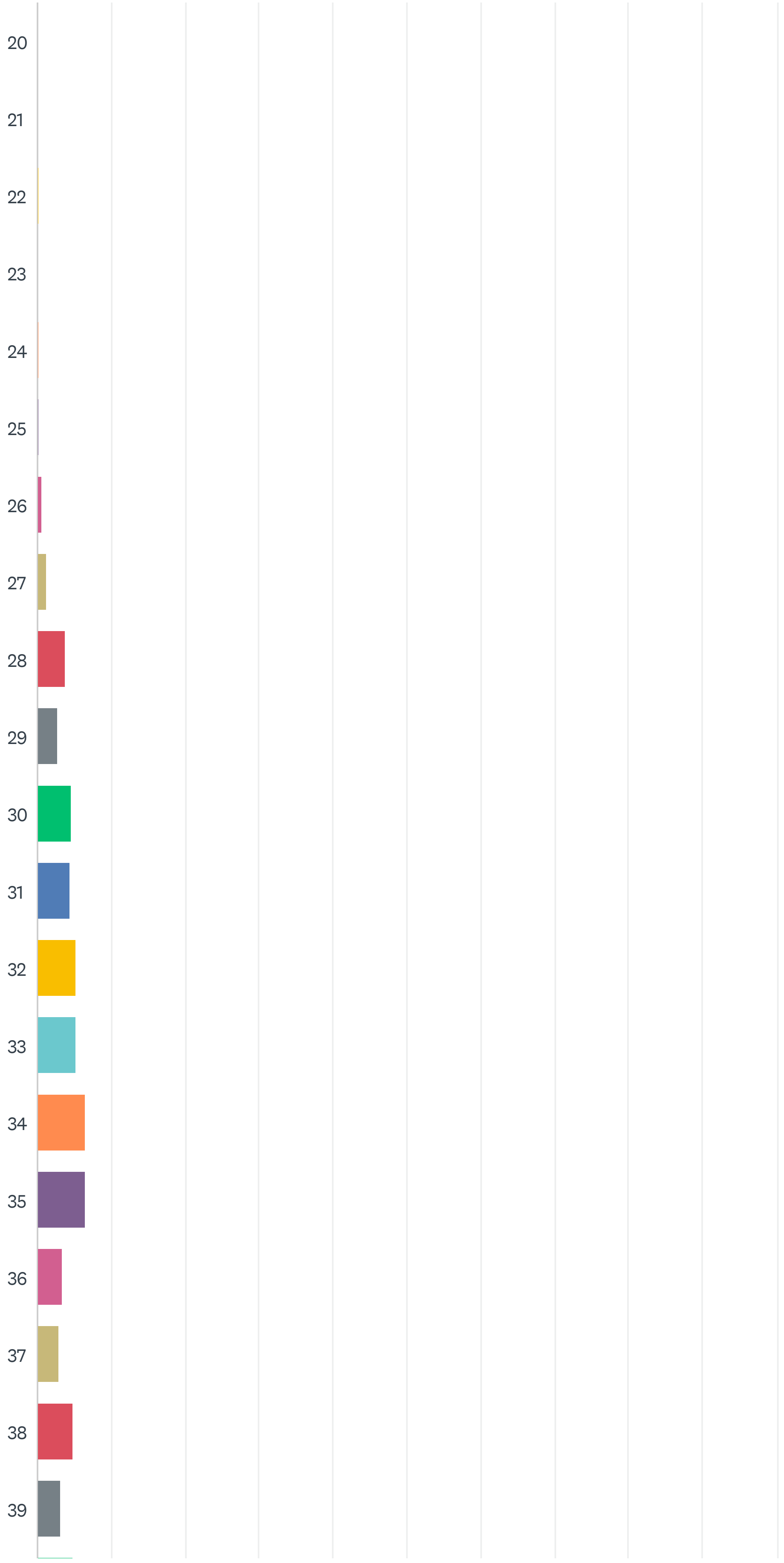

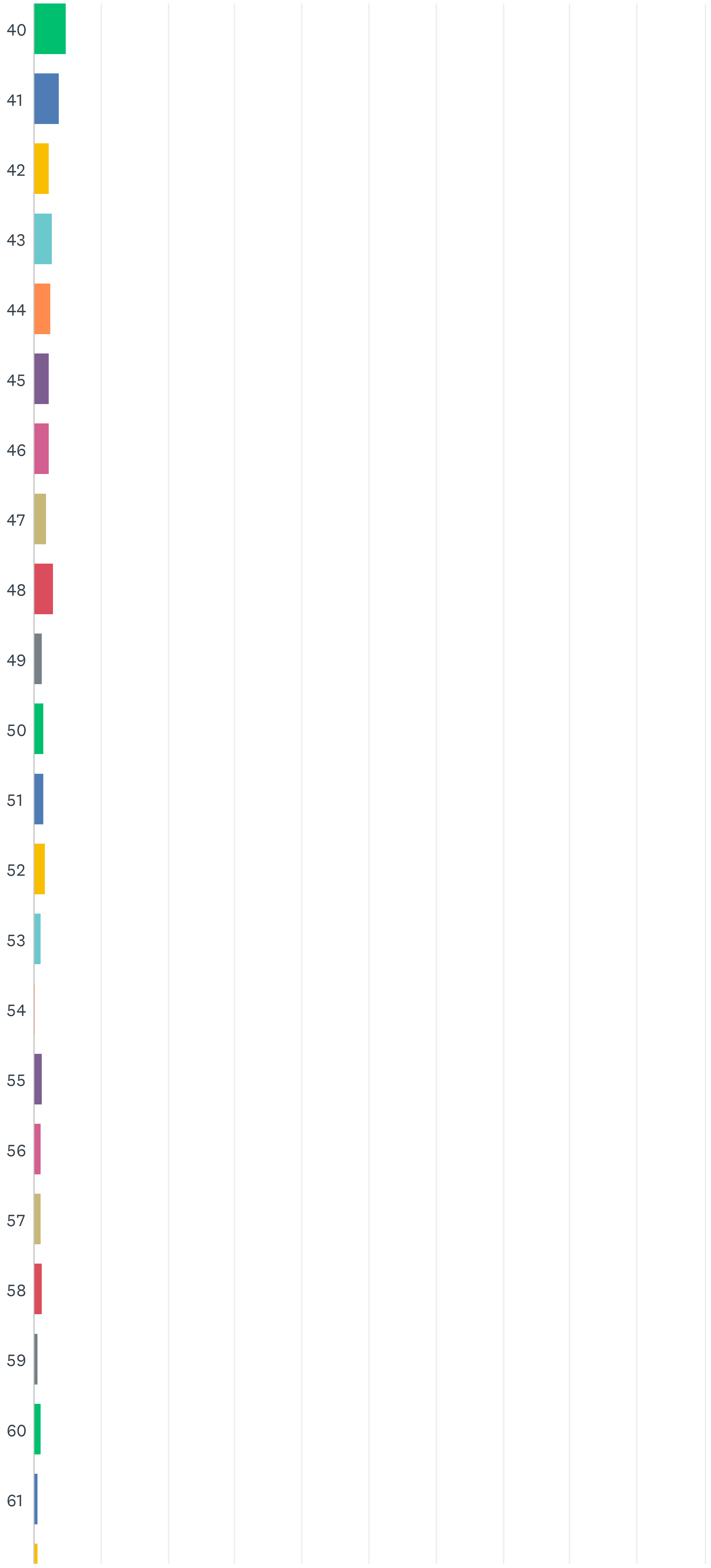
40
41
42
43
44
45
46
47
48
49
50
51
52
53
54
55
56
57
58
59
60
61

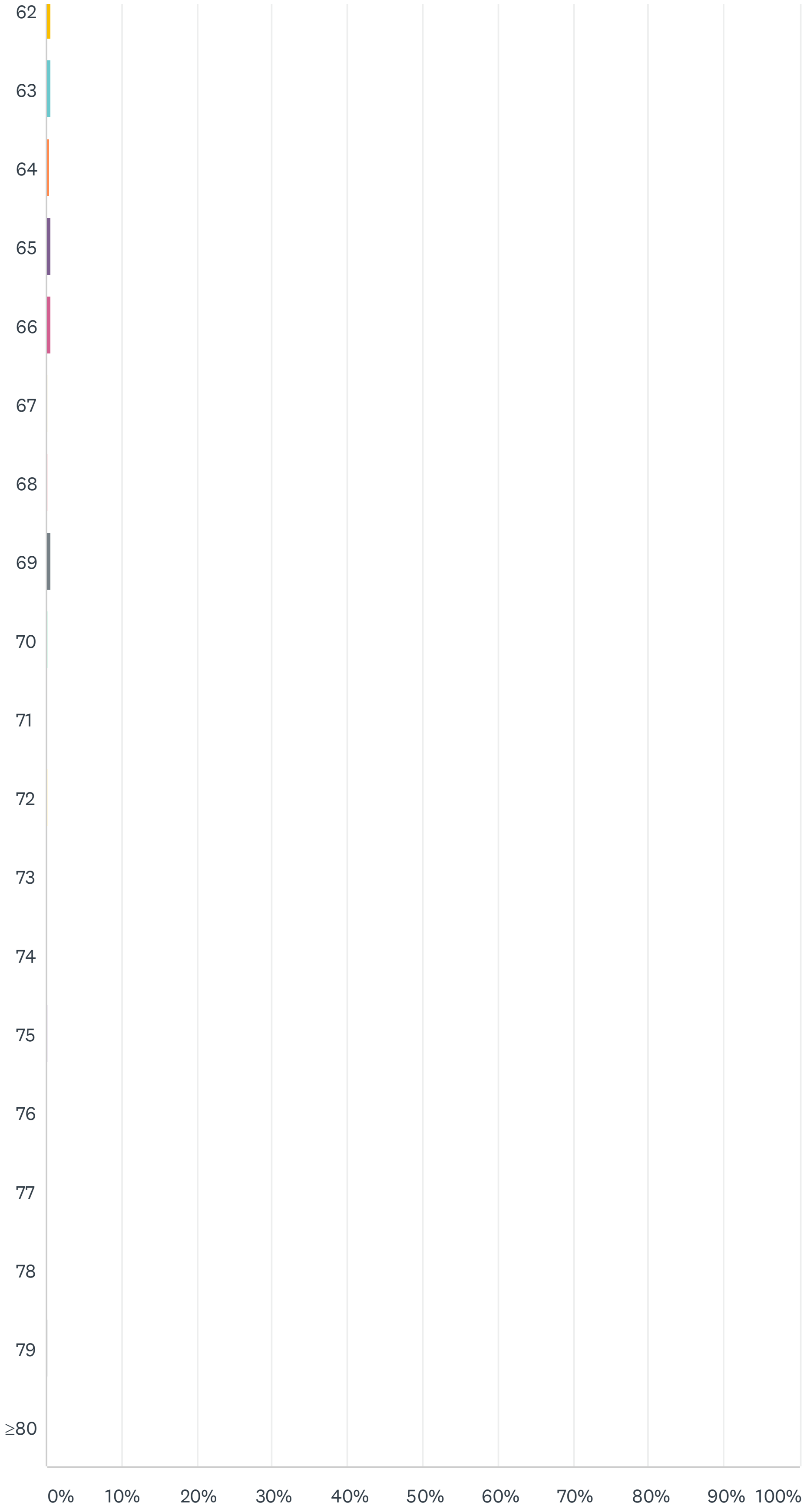
62
63
64
65
66
67
68
69
70
71
72
73
74
75
76
77
78
79
≥80
0%
10%
20%
30%
40%
50%
60%
70%
80%
90%
100%

ANSWER CHOICES	RESPONSES	
20	0.00%	0
21	0.00%	0
22	0.14%	1
23	0.00%	0
24	0.14%	1
25	0.28%	2
26	0.70%	5
27	1.25%	9
28	3.76%	27
29	2.65%	19
30	4.60%	33
31	4.46%	32
32	5.29%	38
33	5.29%	38
34	6.41%	46
35	6.41%	46
36	3.34%	24
37	2.92%	21
38	4.87%	35
39	3.20%	23
40	4.74%	34
41	3.76%	27
42	2.37%	17
43	2.65%	19
44	2.51%	18
45	2.37%	17
46	2.23%	16
47	1.81%	13
48	2.92%	21
49	1.25%	9
50	1.53%	11
51	1.53%	11
52	1.67%	12
53	0.97%	7
54	0.14%	1

55	1.25%	9
56	1.11%	8
57	1.11%	8
58	1.25%	9
59	0.56%	4
60	1.11%	8
61	0.70%	5
62	0.56%	4
63	0.70%	5
64	0.42%	3
65	0.56%	4
66	0.70%	5
67	0.14%	1
68	0.28%	2
69	0.56%	4
70	0.14%	1
71	0.00%	0
72	0.28%	2
73	0.00%	0
74	0.00%	0
75	0.28%	2
76	0.00%	0
77	0.00%	0
78	0.00%	0
79	0.14%	1
≥80	0.00%	0
TOTAL		718

Q36 What is your gender?

Answered: 718   Skipped: 0

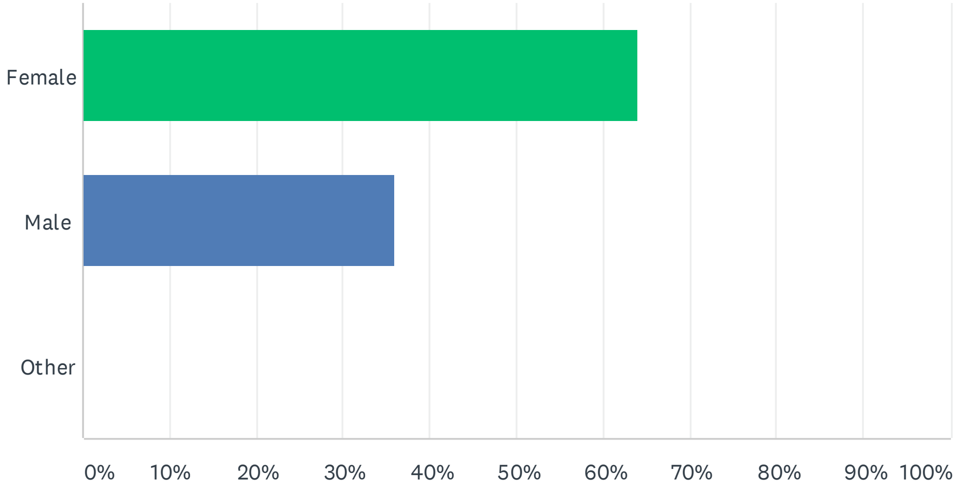

ANSWER CHOICES	RESPONSES	
Female	64.07%	460
Male	35.93%	258
Other	0.00%	0
TOTAL		718

Q37 In what type of practice setting do you mainly work?

Answered: 718   Skipped: 0

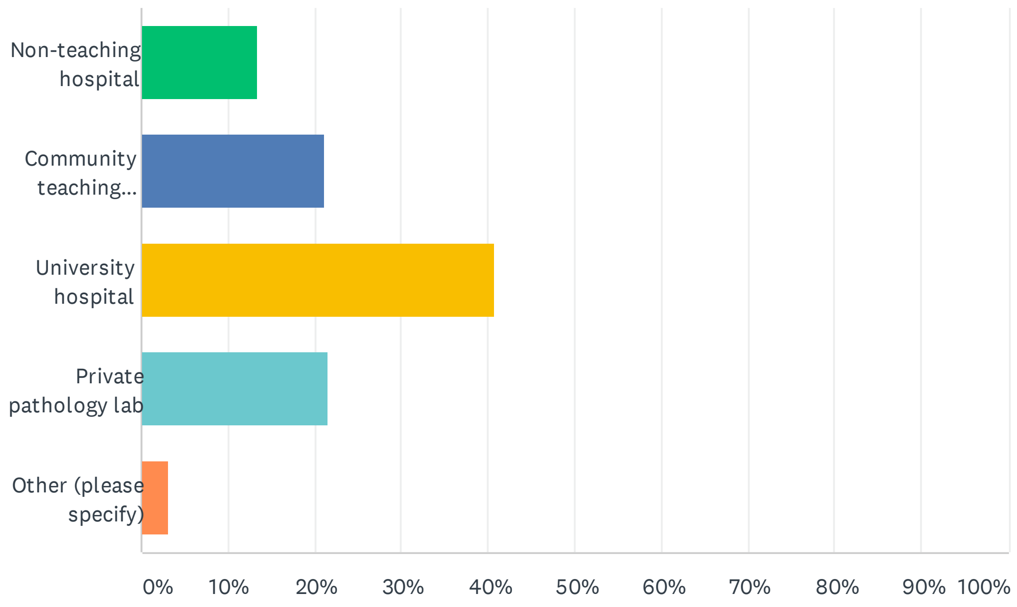

ANSWER CHOICES	RESPONSES	
Non-teaching hospital	13.37%	96
Community teaching hospital	21.17%	152
University hospital	40.81%	293
Private pathology lab	21.45%	154
Other (please specify)	3.20%	23
TOTAL		718

Q38 Which best describes your current position?

Answered: 718   Skipped: 0

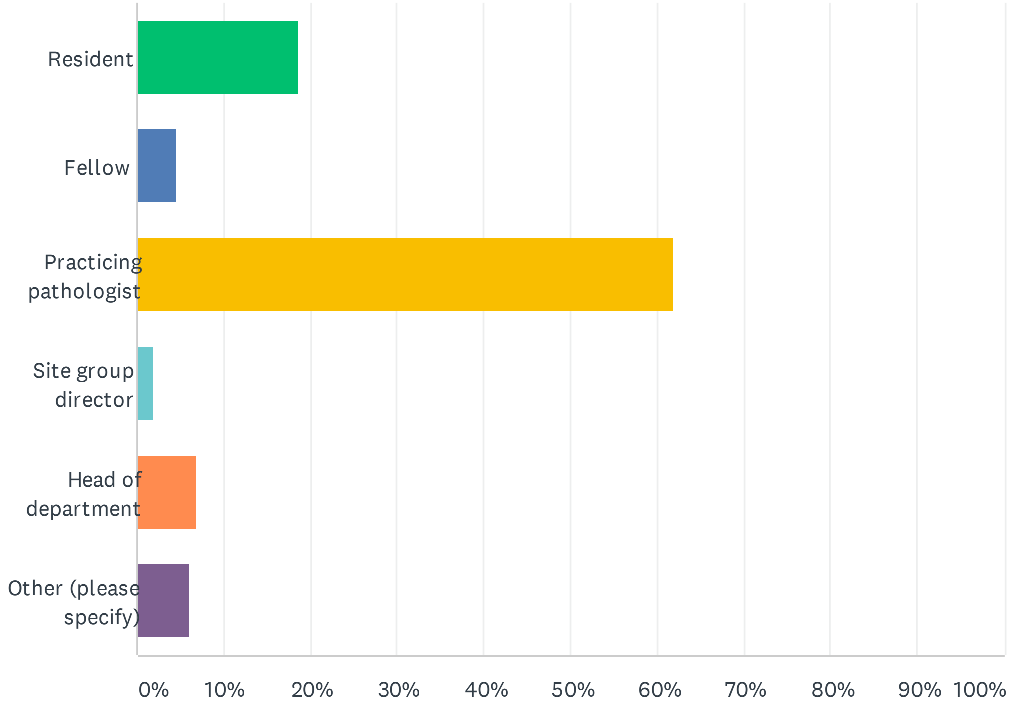

ANSWER CHOICES	RESPONSES	
Resident	18.66%	134
Fellow	4.60%	33
Practicing pathologist	61.84%	444
Site group director	1.95%	14
Head of department	6.96%	50
Other (please specify)	5.99%	43
TOTAL		718
