# Supplementary material for: Attitudes Toward Artificial Intelligence Within Dermatopathology: An International Online Survey
Source: Front Med (Lausanne). 2020 Oct 20;7:591952. doi: 10.3389/fmed.2020.591952 (PMC7606983; doi:10.3389/fmed.2020.591952)
Supplement: Supplementary Table 1 — Distribution of answers to questions regarding background knowledge about AI. Proportions of answers to questions on the specific rows stratified according to access to WSI, previous use of AI in pathology and previous use of AI in dermatopathology. P-values are computed with Fisher's exact test. [file Data_Sheet_3.pdf]

Supplementary Table 1

Question	Yes	Proportion answering Yes to the question			Proportion answering Yes to the question			Proportion answering Yes to the question		
		**Access to WSI**	**No access to WSI**	**P-value**	**Previous use of AI in pathology**	**No previous use of AI in pathology**	**P-value**	**Previous use of AI in dermato-pathology**	**No previous use of AI in dermato-pathology**	**P-value**
AI is a topic that has become of interest for the pathology community. Were you already aware of this topic in pathology?	585 (81.5%)	87.1%	77.7%	**0.001**	93.1%	78.1%	< **0.0001**	91.1%	80.3%	**0.020**
Have you read any medical publications regarding AI within dermatopathology?	181 (25.2%)	34.3%	19.5%	< **0.0001**	38.1%	21.5%	< **0.0001**	59.5%	21.0%	< **0.0001**
Have you used AI as a diagnostic aid in real life within pathology?	160 (22.3%)	31.8%	15.7%	< **0.0001**	-	-	-	81.0%	15.0%	< **0.0001**
Have you used AI as a diagnostic aid in real life within dermatopathology?	79 (11.0%)	18.9%	5.7%	< **0.0001**	40.0%	2.7%	< **0.0001**	-	-	-
		**Proportion with Good or excellent knowledge**			**Proportion with Good or excellent knowledge**			**Proportion with Good or excellent knowledge**		
	**Good or excellent knowledge**	**Access to WSI**	**No access to WSI**	**P-value**	**Previous use of AI in pathology**	**No previous use of AI in pathology**	**P-value**	**Previous use of AI in dermato-pathology**	**No previous use of AI in dermato-pathology**	**P-value**
Which degree of knowledge would you say you have when it comes to AI within pathology?	135 (18.8%)	28.2%	12.1%	< **0.0001**	31.3%	15.2%	< **0.0001**	45.6%	15.5%	< **0.0001**

Abbreviations: AI, artificial intelligence; WSI, whole slide imaging.

Supplementary Table 2

Question	Agree or strongly agree	Proportion that agree or strongly agree to the question			Proportion that agree or strongly agree to the question			Proportion that agree or strongly agree to the question		
		Access to WSI	No access to WSI	P-value	Previous use of AI in pathology	No previous use of AI in pathology	P-value	Previous use of AI in dermato-pathology	No previous use of AI in dermato-pathology	P-value
AI will revolutionize Medicine in general.	534 (74.4%)	76.3%	75.5%	0.86	81.0%	74.3%	0.092	81.6%	75.1%	0.26
AI will revolutionize dermatopathology.	435 (60.6%)	59.3%	64.4%	0.20	67.7%	61.0%	0.13	74.7%	61.0%	**0.023**
AI will revolutionize dermatopathology more than other subfields within pathology.	133 (18.5%)	20.0%	19.2%	0.84	23.7%	18.5%	0.17	42.7%	16.8%	**< 0.0001**
In the foreseeable future all physicians will be replaced by AI.	43 (6.0%)	7.2%	5.1%	0.25	12.9%	4.2%	**0.0002**	27.0%	3.6%	**< 0.0001**
The human pathologist will be replaced by AI in the foreseeable future.	44 (6.1%)	6.2%	6.7%	0.87	11.0%	5.0%	**0.013**	23.0%	4.3%	**< 0.0001**
A development with an increased use of AI in dermatopathology frightens me.	116 (16.2%)	14.6%	17.6%	0.35	16.3%	16.1%	1	21.5%	15.5%	0.19
A development with an increased use of AI in dermatopathology makes dermatopathology more exciting to me.	427 (59.5%)	57.1%	61.0%	0.31	64.4%	58.1%	0.17	69.6%	58.2%	0.053
A development with an increased use of AI makes medicine in general more exciting to me.	464 (64.6%)	62.9%	65.8%	0.47	66.3%	64.2%	0.64	75.9%	63.2%	**0.025**
AI will improve dermatopathology	519 (72.3%)	75.6%	75.8%	1	77.9%	74.9%	0.52	86.3%	74.3%	**0.022**

AI will improve medicine in general.	580 (80.8%)	84.1%	83.2%	0.83	83.0%	83.4%	0.90	85.3%	83.1%	0.74
AI should be part of medical training.	604 (84.1%)	84.4%	87.8%	0.21	87.8%	85.7%	0.60	90.9%	85.6%	0.23
I consider myself well-informed about the use of modern technology, especially computers.	534 (74.4%)	81.8%	68.9%	**0.0001**	79.4%	72.9%	0.12	79.7%	73.7%	0.28

Abbreviations: AI, artificial intelligence; WSI, whole slide imaging
